# How filopodia respond to calcium in the absence of a calcium-binding structural protein: non-channel functions of TRP

**DOI:** 10.1186/s12964-022-00927-y

**Published:** 2022-08-26

**Authors:** C. A. Heckman, O. M. Ademuyiwa, M. L. Cayer

**Affiliations:** 1 Department of Biological Sciences, 217 Life Science Building, Bowling Green State University, Bowling Green, OH 43403-0001 USA; 2 Center for Microscopy and Microanalysis, Bowling Green State University, Bowling Green, OH 43403 USA

**Keywords:** Cell signaling, Rapidly releasable pool, Transient receptor potential channel, Directional persistence, Stromal-interacting molecule, Plus-end tracking proteins

## Abstract

**Background:**

For many cell types, directional locomotion depends on their maintaining filopodia at the leading edge. Filopodia lack any Ca^2+^-binding structural protein but respond to store-operated Ca^2+^ entry (SOCE).

**Methods:**

SOCE was induced by first replacing the medium with Ca^2+^-free salt solution with cyclopiazonic acid (CPA). This lowers Ca^2+^ in the ER and causes stromal interacting molecule (STIM) to be translocated to the cell surface. After this priming step, CPA was washed out, and Ca^2+^ influx restored by addition of extracellular Ca^2+^. Intracellular Ca^2+^ levels were measured by calcium orange fluorescence. Regulatory mechanisms were identified by pharmacological treatments. Proteins mediating SOCE were localized by immunofluorescence and analyzed after image processing.

**Results:**

Depletion of the ER Ca^2+^ increased filopodia prevalence briefly, followed by a spontaneous decline that was blocked by inhibitors of endocytosis. Intracellular Ca^2+^ increased continuously for ~ 50 min. STIM and a transient receptor potential canonical (TRPC) protein were found in separate compartments, but an aquaporin unrelated to SOCE was present in both. STIM1- and TRPC1-bearing vesicles were trafficked on microtubules. During depletion, STIM1 migrated to the surface where it coincided with Orai in punctae, as expected. TRPC1 was partially colocalized with Vamp2, a rapidly releasable pool marker, and with phospholipases (PLCs). TRPC1 retreated to internal compartments during ER depletion. Replenishment of extracellular Ca^2+^ altered the STIM1 distribution, which came to resemble that of untreated cells. Vamp2 and TRPC1 underwent exocytosis and became homogeneously distributed on the cell surface. This was accompanied by an increased prevalence of filopodia, which was blocked by inhibitors of TRPC1/4/5 and endocytosis.

**Conclusions:**

Because the media were devoid of ligands that activate receptors during depletion and Ca^2+^ replenishment, we could attribute filopodia extension to SOCE. We propose that the Orai current stimulates exocytosis of TRPC-bearing vesicles, and that Ca^2+^ influx through TRPC inhibits PLC activity. This allows regeneration of the substrate, phosphatidylinositol 4,5 bisphosphate (PIP2), a platform for assembling proteins, e. g. Enabled and IRSp53. TRPC contact with PLC is required but is broken by TRPC dissemination. This explains how STIM1 regulates the cell’s ability to orient itself in response to attractive or repulsive cues.

**Video Abstract**

**Supplementary Information:**

The online version contains supplementary material available at 10.1186/s12964-022-00927-y.

## Background

The development and differentiation of a tissue depends on the cells’ mechanisms for determining polarity. Degradation of these mechanisms has profound implications for epithelial lining tissues, where the loss of polarity is recognized as an early sign of neoplasia. In many cell types in vitro, the dynamics of filopodia assembly and disassembly are essential for maintaining polarity. This is also true of cells in vivo. In endothelial tip cells, filopodia at the leading edge increase the efficiency of cell advancement during angiogenic sprouting [87]. They function similarly in the neuronal growth cone where they mediate guidance, i.e. the directional changes needed for efficient axon navigation through nonneuronal tissues. The receptors for vascular endothelial growth factor (VEGF), VEGFR2, and VEGFR3 are localized on filopodia of the tip cell, and its directional locomotion is guided by the gradient of VEGF [27, 99]. Likewise, receptors for insulin-like growth factor 1 and epidermal growth factor (EGF) are concentrated on filopodia [50, 54]. The receptors themselves may be internalized into the cell through a clathrin-coated pit at the base of the filopodium [102]. Whereas these signals must be interpreted in the context of signaling from substrate adhesion, it is not known how they are mixed and made meaningful to the cell.

The SOCE process has also been implicated in polarity determination. SOCE is the process by which the depletion of calcium stores in the ER causes influx of Ca^2+^ across the plasma membrane. It is stimulated by the release of intracellular calcium from the ER downstream of receptor ligation. The Ca^2+^ efflux is triggered by inositol 1,4,5 trisphosphate (IP_3_), which stimulates the opening of a Ca^2+^ channel in the ER called IP_3_ receptor (IP_3_R). The reduced Ca^2+^ concentration in the ER lumen activates the Ca^2+^ sensor, stromal interaction molecule (STIM). Membrane vesicles containing STIM associate with adenomatous polyposis coli and end-binding proteins. These plus-end, microtubule-associated proteins mediate the translocation of ER fragments to the plasma membrane [4, 32]. STIM forms a complex with the Orai channel and activates Orai, which then admits Ca^2+^ into the cell. This is the Ca^2+^ release-activated Ca^2+^ (CRAC) current which is identified by its electrophysiological signature (see for reviews [14, 97]). Although Orai1 and STIM are indispensable components of SOCE, TRP channels are often activated downstream resulting in a nonspecific cation influx into the cell (see for review [22]). TRP channels are thought to amplify the Ca^2+^ flux that was initiated by the activation of Orai, but they are also linked to cytoskeleton (see for review [96]).

Previous studies suggested that both the TRPC channels and the sensory protein, STIM, affected filopodia dynamics. These genes were essential for axon guidance, and the attractive turning of the growth cone could be switched to repulsive turning by knock-down of either one [69, 94, 113]. Genetic ablation of TRPC1 also prevented the formation of filopodia on the tip cells during sprouting angiogenesis [120]. Thus, previous work suggested that some aspect of SOCE was implicated in filopodia formation in several cell types (see for review [1, 26]). The complexity of the process has made it difficult to dissect the relationship, but it has been proposed that filopodia respond to Ca^2+^ influx through TRP channels. Reports on growth cones showed that filopodia extension follows an elevation of [Ca^2+^]_i_ but other studies, detailing filopodia responses to spontaneous Ca^2+^ transients in dendrites, showed the contrary (see for review [1]). In the latter experimental model, low intracellular Ca^2+^ was required to initiate extension [58]. One fundamental obstacle to TRP-mediated regulation of filopodia dynamics is the absence of any Ca^2+^-mediated mechanism for their extension. In muscle cells, this role is filled by calponin C, a protein that changes conformation upon Ca^2+^ binding. This displaces tropomyosin from the actin filament, a mechanism that is essential to initiate contraction. The structural constituents of filopodia are well-known, and there is a marked absence of Ca^2+^-binding proteins [23, 24, 37, 42, 64]. There is a more circuitous relationship between intracellular Ca^2+^ concentrations and filopodia, however. Macropinocytosis, which is triggered by Ca^2+^ release from ryanodine-sensitive internal stores, causes filopodia disappearance and growth cone retraction [44].

The above considerations argued against a direct effect of Ca^2+^ influx through TRP channels. Nevertheless, there is evidence suggesting a relationship between the SOCE process and filopodia. In several experimental systems, patterns of TRPC and STIM trafficking were subject to regulation by phosphoinositide 3-kinase (PI3-K) and PTEN (Phosphatase and TENsin homolog deleted on chromosome 10). This suggested an interaction of SOCE mediators with determinants of polarity [16, 49, 70]. The PI3-K/PTEN axis is a highly conserved mechanism for maintaining the cell’s anterior–posterior axis, and SOCE mediators may respond to it by directional trafficking to the leading edge. This would mean SOCE affected polarity indirectly by affecting filopodia. These considerations are complicated by the complexity of Ca^2+^ signaling, because its concentration in cells undergoes fluctuations on both global and local scales. In migrating cells, the Ca^2+^ concentration is low at the leading edge and high at the rear of the cell. Within the lowest portion of this gradient, calcium flickers were found [101], which are sites where IP_3_R is activated [116].

In order to clarify these relationships, we attempted to discover how filopodia were affected by SOCE mediators. The Ca^2+^ concentration in the ER is many-fold higher than in the cytoplasm. Lowering it by efflux through IP_3_R initiates the SOCE process. Here, we removed exogenous stimuli and then broke SOCE down into two phases, namely ER depletion and readdition of Ca^2+^. This allowed us to analyze filopodia prevalence as a function of intracellular calcium, [Ca^2+^]_i_ and Ca^2+^ influx. The results showed that STIM and TRPC1 underwent redistribution within the cells during each phase. All the distributions of SOCE mediators differed from those of an aquaporin channel unrelated to SOCE, AQP4. AQP4, an aquaporin characteristic of respiratory airway lining cells, shared compartments with both TRPC1 and STIM1. Its trafficking pattern shared properties with both as well. TRPC1 was not confined to filopodia after Ca^2+^ readdition, but its translocation to the cell surface was closely related to filopodia dynamics. TRPC exocytosis appeared to depend on the influx of extracellular Ca^2+^ through the Orai channel.

## Materials and methods

### Cell culture and treatment with pharmacological agents

An immortalized rat cell line, 1000 W, was used. It was originally generated from rat tracheal epithelium as a model of human bronchogenic carcinoma [62]. The cells were maintained in a modified Waymouth's medium (Sigma-Aldrich, St. Louis, MO) containing penicillin, streptomycin, 10% fetal bovine serum (Hyclone, UT or Atlanta Biologicals, GA), 0.1 µg/ml insulin, and 0.1 µg/ml hydrocortisone, as previously described [36]. They were subcultured by detachment with a trypsin solution (Invitrogen, Grand Island, NY) made up in Ca^2+^-, Mg^2+^-free Hanks’ balanced salt solution (Ca^2+^-free HBSS, GIBCO, Gaithersburg, MD).

To inhibit TRPC channels, SKF96365 (1-(beta-[3-(4-methoxy-phenyl)propoxy]-4-methoxyphenethyl)-1H-imidazole hydrochloride) was obtained from Selleckchem (Munich, Germany). SKF96365 blocked nonselective cation channel activity with IC50s of 3 to 16 µM [35, 66]. Pico145 (Chem Scene, Monmouth Junction, NJ) was used at 0.3–1.3 nM to inhibit TRPC1/4/5 isoforms. Nifedipine (MedChem Express, Monmouth Junction, NJ) was used to inhibit voltage-activated calcium channels (VACC). The reagents were used at final concentrations of 1–3 times the IC50 for the respective activities [66, 91], except for CRAC and VACC channel inhibitors. These inhibitors were active against the respective channels at micromolar concentrations [115, 125], but higher concentrations were needed to affect filopodia.

Possible mediators of the response to altered [Ca^2+^]_i_ were investigated by using cell-permeable peptides, enzyme inhibitors, and receptor agonists. 1,2-dioctanoylglycerol (DOG) was obtained from Sigma-Aldrich, and the aldehyde inhibitor of calpains, ALLN, from Focus Biomolecules (Plymouth, PA). Calcium-like peptide 2 (CALP2) and N-(6-aminohexyl)-5-chloro-1-naphthalenesulfonamide hydrochloride (W-7) were obtained from Tocris (Minneapolis, MN). CaM kinase II and calcineurin inhibitors, autocamtide-2-related inhibitory peptide and calcineurin autoinhibitory peptide were obtained from EMD Millipore, Temecula, CA. Myosin light chain kinase (MLCK) inhibitors, ML-7 and MLCK peptide, were obtained from Cayman Chemicals (Ann Arbor, MI). When epidermal growth factor (EGF) was used to supplement Ca^2+^-free HBSS, the human recombinant protein (PeproTech, Rocky Hill, NJ) was used at a final concentration of 10 ng/ml.

### Sample preparation, filopodia counts, and statistical analysis

Cells were fixed with warm, buffered 3% formaldehyde (pH 7.4) made fresh from paraformaldehyde in cytoskeletal buffer. The samples were rinsed with phosphate-buffered saline and stored in buffer at 4 °C until observations were made. Samples were mounted on slides and assigned code numbers before being examined, and counts were made by independent observers who had no knowledge of the sample’s identity. Filopodia prevalence was determined on single cells by analyzing the fraction of cells with filopodia and the proportion of their perimeter covered with filopodia. The morphology of the filopodia was the same as shown elsewhere [36], and determinations were based on the quantitative methods of deconstructing protrusions used previously. Because the coverage of the cell perimeter varied from one experiment to another, counts are presented relative to counts in the sham-treated control or counts at the starting point before Ca^2+^ readdition [36]. Microsoft Excel was used to calculate averages and standard deviations.

An online service, https://www.statskingdom.com/320ShapiroWilk.html, was used to test the variability of filopodia measurements. The control sample means, tested for 20 experiments, showed a probability value of *P* = 0.342. This indicated a low probability that the populations deviated from the normal distribution. Differences among the means of experimental values were evaluated using the online service for one-way ANOVA, https://www.socscistatistics.com/tests/anova/default2.aspx. The Tukey test was used for multiple comparisons. Comparisons between individual treatment groups and controls were done by the two-tailed Student’s t-test with Bonferroni correction for multiple comparisons. All error bars shown represent ± one standard error of the mean (S.E.M.)

### Germanium substrates for cell culture

To make the substrates adhesive for 1000 W cells, a film of germanium (Structure Probe, Inc., West Chester, PA) was applied to glass coverslips of 25 mm diameter and thickness #1 (Electron Microscopy Sciences, Hatfield, PA) as previously described [36]. Coverslips were sterilized by ultraviolet irradiation and placed in 35-mm culture dishes. For experiments, 2–3 × 10^5^ cells were plated in each 35-mm dish and left overnight to become attached.

### Calcium store depletion and replenishment

To deplete the ER calcium store, the culture medium was replaced with Ca^2+^-free HBSS containing 1.5 µM ethylene glycol-bis(β-aminoethyl ether)-N,N,N',N'-tetraacetic acid (EGTA) or 5 µM cyclopiazonic acid (CPA, CalBiochem-EMD Millipore). For Ca^2+^ replenishment, the coverslips with the cells attached were transferred sequentially into Ca^2+^-free HBSS for CPA washout (5 min) and then Ca^2+^-replete Hanks balanced salt solution (HBSS). Although the effects of CPA and EGTA on filopodia were indistinguishable during store depletion, recovery was more reproducible with CPA.

Filopodia only increased if the CPA treatment time was extended to 30 min (see “Ca^2+^ depletion by CPA causes a gradual rise in [Ca^2+^]_i_ and transient filopodia formation” section). We used 30 min as the standard interval for depleting the ER of Ca^2+^ except in experiments where we were testing for accelerated depletion. After Ca^2+^ readdition, filopodia increased for 20 min (data not shown), but their prevalence initially showed high variability. As the recovery of ER Ca^2+^ depended on washing out CPA, a single time was set for collecting cells. This minimized variability after Ca^2+^addition (see “Changes in filopodia prevalence during the two-step procedure” section).

### Changes in Ca^2+^ concentration

Intracellular calcium levels were compared in cells incubated in Ca^2+^-free HBSS before ER stress and during depletion of the ER with CPA. This was done by exposing cells to calcium orange AM (Invitrogen, Eugene, OR) for 20 min in culture medium, then transferring the cells to Ca^2+^-free HBSS with or without CPA. Calcium orange was made up at 0.25 µg/µl in dimethylsulfoxide and used at a final concentration of 2 µg/ml. Images were acquired at 5-min intervals, using the software, instrumentation and settings described below (see “Equipment and settings” section) and processed by background subtraction. The averaged intensity value per cell was calculated for each frame processed.

### Immunofluorescence localization, image acquisition, and image processing

For immunofluorescence staining, cells were fixed as above and permeabilized with 50 µg/ml digitonin (LC Laboratories, Woburn, MA) and 0.2% Triton-X 100 in cytoskeletal buffer. Phospholipase C isoform (PLCβ2, MyBioSource.com) and antibodies against Orai1 and Orai3 (ProSci, Poway, CA) were made in rabbit. Mouse monoclonal antibodies included anti-β-tubulin and rabbit polyclonal anti-TRPC1 from Sigma-Aldrich and antibodies against Vamp2 (R&D Systems, Minneapolis, MN), CaV1.2 (Novus Biologicals, Centennial, CO), caveolin-2 (Thermo Fisher, Rockford, IL), β1 integrin (BD Biosciences Pharmingen), STIM1/CRACR2A (CRAC regulator 2A, Cedarlane Laboratories, Burlington NC), and a combination of five clones against PLCγ1 (Sigma-Aldrich).

In experiments with sheep antibody against the extracellular loop of TRPC1 (Antibodies-online, Limerick, PA), primary staining was followed by Cyanine 3-labeled donkey anti-sheep antibody from Jackson ImmunoResearch (West Grove, PA). Where a rabbit and mouse primary were used, secondary staining was performed using donkey tetra-rhodamine isothiocyanate (TRITC)-labeled anti-mouse IgG and goat fluorescein isothiocyanate (FITC)-conjugated anti-rabbit antibodies (Jackson ImmunoResearch). Triple staining procedures were done using the secondary antibodies, donkey anti-mouse (Abcam, Cambridge, MA) tagged with Cyanine5, Cyanine 3-tagged anti-sheep, and FITC-tagged anti-rabbit. Samples were mounted in 2.5% DABCO made up in 2,2′-thiodiethanol (Sigma-Aldrich) and viewed with a 100 × lens as described below (see “Equipment and settings” section). Control samples that were unexposed or exposed to irrelevant antibodies, followed by the secondary antibodies, showed no staining.

### Equipment and settings

To assess intracellular Ca^2+^ concentration, we used a confocal Leica DMI3000B inverted microscope (Leica Microsystems, Buffalo Grove, IL) equipped with a Lumen Dynamics X-Cite light engine and Leica rhodamine filter set (Excitation 546/10 nm, dichroic LP 560 nm, Emission 585/40 nm) and 10 × lens. Images were acquired once per minute with a Rolera Thunder cooled CCD camera with back-thinned, back-illuminated, electron-multiplying sensor (QImaging, Surrey, British Columbia, Canada). MetaMorph version 7.8, 4.0, (Molecular Devices, Sunnyvale, CA) was used to configure the hardware settings for time-lapse recording.

Samples triple-stained for the SOCE mediators, STIM1, Orai1/3, and TRPC1, were also imaged with the Leica DMI3000B inverted microscope using Spectra X LED source (Lumencor, Beaverton, OR) and X-Light spinning-disk confocal unit (CrestOptics, Rome, Italy) with Semrock bandpass filter FF01-440/521/607/700–25 and dichroic FF410/504/582/669-Di0l-25 × 36. The software and CCD camera used were those identified above.

For studies by higher magnification, epifluorescence images were taken on an Axiophot microscope (Carl Zeiss, Jena) using Zeiss 100 × Plan-Neofluar/1.30 objective lens and FluoArc mercury lamp. Filter sets for FITC (excitation 490 nm, emission 525 nm) and Cyanine3 or TRITC (excitation 545 nm, emission 605 nm) were from Chroma (Bellows Falls, Vermont). Images were acquired with an Andor camera (Zyla 4.2 PLUS sCMOS, Concord, MA) running under Molecular Devices MetaVue software. The final pixel size was 63 nm.

### Refractive index determination

To determine the role of water entry in filopodia formation, bovine serum albumin (Fisher Fraction V, Heat Shock Treated) was made up in Waymouth’s medium (see “Cell culture and treatment with pharmacological agents” section) at 33.35% (w/v) and diluted 3:4 with medium before use. Cells were cultured on germanium coverslips and maintained at 37 °C while being viewed. Time-lapse recordings were made using a Plan-apochromatic 100 × lens in the Zeiss Axiophot microscope, with image acquisition as described above (see “Equipment and settings” section).

### Correlation coefficients

Pearson correlation coefficients were obtained for matched images of thin, peripheral areas viewed in the FITC and tetra-rhodamine channels. The Colocalization Finder [53] plugin, running under ImageJ [89], was used. With this plugin, background outside the cells and thicker parts of the interior could be excluded from analysis. Because we did not attempt to remove the background, the coefficients included fluorescence background surrounding the particles. This was nonspecific, so a value around 0.4 was the lowest average coefficient observed.

### Analysis of particle sizes after staining by indirect immunocytochemistry

The different loci, represented by immunocytochemical localizations of their intracellular sites, were segmented from images of thin, peripheral portions of the cells. The selection of these areas obviated the complication of multiple layers of structures, as overlapping layers of particles were absent. Contrast enhancement and edge detection operations were run on each image in ImageJ. Then, the threshold was adjusted to maximize the number of continuous outlines around figures. The figures’ sizes were measured in pixels, using the particle analysis plugin and a circularity setting of 0.9–1.0. The radius was calculated by modeling the number of pixels in each locus as a circle, and the relative size distributions were compared on plots. As the images used to generate the data were partial images of cells, and some cells lacked areas suitable for particle analysis, the number of images subjected to analysis was used as the sample number in statistical tests.

To determine the diameter of the zone occupied by TRPC1 clusters at the cell surface, we estimated the maximum extent of the zone around each filopodium using the line function of ImageJ. The analysis was conducted around the zone of each filopodium where the loci were clearly in-focus. Loci, referred to as particles here, were assigned to the maximum distance, and the results were plotted as a cumulative distribution of distances.

## Results

### Changes in filopodia prevalence during the two-step procedure

**Fig. 1:**
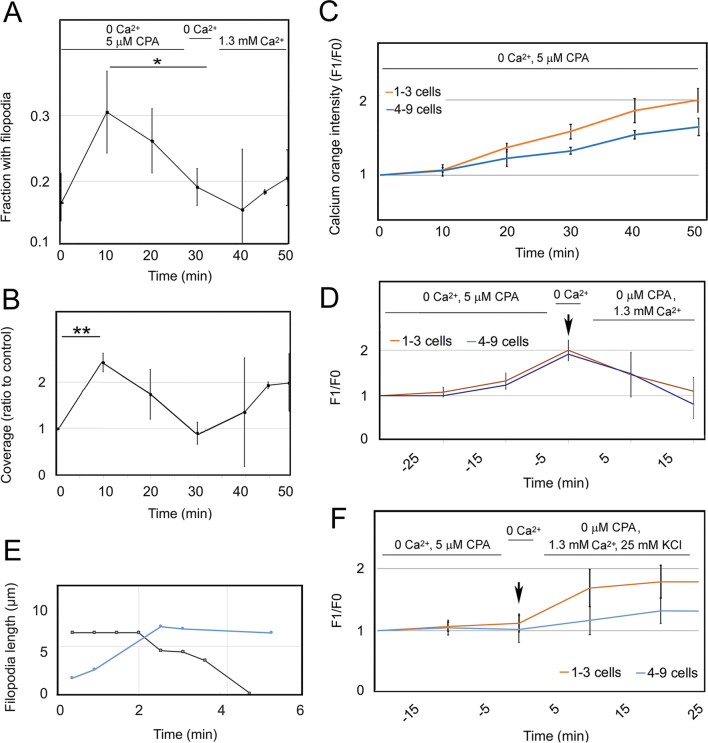
[Ca^2+^]_i_ either low and rising or high and falling is permissive for filopodia. Cells were treated with 5 µM CPA or 1.5 µM EGTA in Ca^2+^-free HBSS to deplete the ER of Ca^2+^. Results in panels A-C and E-F represent 3–7 experiments. (A, B) ER depletion followed by CPA washout and readdition of extracellular Ca^2+^. At various times, samples were collected and the fraction of cells with filopodia and percent coverage of the perimeter determined. **a** Significance by ANOVA, *P* = 0.0017. *Treatments differ at *P* = 0.0015. **b** Significance by ANOVA, *P* = 0.0030. **Treatments differ at *P* = 0.0028. **c** Fluorescence intensity of groups of cells preloaded with calcium orange and exposed to Ca^2+^-free HBSS with CPA. Areas with 1–3 cells and colonies of 4–9 cells are analyzed separately, and emission is shown as the ratio of the intensity over initial intensity (F_1_/F_0_). Significance of experiment by ANOVA repeated measures, *P* < 0.0001. **d** Fluorescence intensity of calcium orange during ER depletion and Ca^2+^ readdition. Depletion is represented by negative numbers, and Ca^2+^ readdition is at time = 0 (arrow). The means, tested by ANOVA repeated measures, differed significantly at *P* = 0.0042. **e** Filopodia extension and retraction. Typical extension (blue) and retraction (black) rates are 1.7 µm/min and 1.4 µm/min, respectively. The mean length was 5.7 µm (S.D. 1.0 µm, filopodia in 8 images). **f** Increase in F_1_/F_0_ after CPA washout followed by Ca^2+^ with 25 mM KCl, supplied at time = 0 (arrow). Values after time = 0 differed significantly by the ANOVA repeated measures test, *P* < 0.0001. Error bars represent ± one standard error of the mean (S.E.M.)

A classical procedure was used to separate the SOCE process into two phases [79]. In the first step, cells were deprived of Ca^2+^ in the presence of CPA. It is well-established that this depletes the ER of Ca^2+^ and causes the formation of a STIM1-Orai complex at the plasma membrane. Ca^2+^ deprivation increased the filopodia briefly. This was followed by a spontaneous decline to the starting level by 30 min. In a second step, refilling of the ER is initiated by CPA washout and replacement of the Ca^2+^-free medium with Ca^2+^-replete medium. After Ca^2+^ readdition, the prevalence of filopodia rose (Fig. 1a, b), and it remained high for longer than the duration of a typical cycle of extension and retraction (see “[Ca^2+^]_i_ either low and rising or high and falling is permissive for filopodia” section). To determine the effect of CPA in Ca^2+^-replete solution, we conducted the experiment with Ca^2+^ present at all stages. This had a negligible effect on the number of cells with filopodia (Additional file 1: Fig. 1A). The percentage of the edge covered with filopodia increased but did not change significantly afterwards (Additional file 1: Fig. 1B). By introducing standard rinse procedures before and after ER depletion, we got reproducible increases in filopodia during replenishment (see “Filopodia formed during Ca^2+^ replenishment depend on TRPC1/4/5” section and Fig. 7c, d).

#### Ca^2+^ depletion by CPA causes a gradual rise in [Ca^2+^]_i_ and transient filopodia formation

To determine how filopodia formation was related to [Ca^2+^]_i_, we preloaded cells with a Ca^2+^ indicator and visualized the Ca^2+^-dependent fluorescent emission by confocal microscopy. Merely replacing the culture medium with Ca^2+^, Mg^2+^-free balanced salt solution caused little change in emission over 30 min (see Additional file 1: Fig. 2). This was consistent with previous results showing that intracellular Ca^2+^ concentrations were not affected by lowering extracellular Ca^2+^ levels [20]. In contrast, Ca^2+^ deprivation with CPA caused [Ca^2+^]_i_ to increase for 40 min (Fig. 1c). This was attributed to inhibition of the sarcoplasmic reticulum Ca^2+^-ATPase (SERCA) pump by CPA, which prevented Ca^2+^ being returned to the ER. After 15 min of CPA treatment, [Ca^2+^]_i_ began to increase, while filopodia declined (Fig. 1a–c). However, replacing culture medium with balanced salt solution also resulted in removal of all of the growth factors and inhibitors ordinarily supplied in fetal bovine serum. This altered downstream pathways, such as signaling to IP_3_R through IP_3_-mediated calcium release, as illustrated in Additional file 1: Fig. 3A. Testing for its effects, we found that substitution alone had little effect on the number of cells showing filopodia (Additional file 1: Fig. 3B). The percentage of the periphery showing filopodia showed a slight increase. This was observed in all experiments after medium substitution but was only statistically significant when the treatment caused ER depletion (cf. Fig. 1b and Additional file 1: Figs. 1 and 3C).

#### [Ca^2+^]_i_ either low and rising or high and falling is permissive for filopodia

When CPA was washed out and extracellular Ca^2+^ was restored, [Ca^2+^]_i_ decreased again (Fig. 1d). This was the expected result, because the removal of CPA relieved inhibition of the SERCA transporter, enabling Ca^2+^ to be pumped back into the ER (see for review [78]). During this part of the two-step procedure, filopodia increased again while [Ca^2+^]_i_ decreased to the level of untreated cells (cf. Fig. 1a, b, d). To determine whether elevating the [Ca^2+^]_i_ further would result in an even greater depression of filopodia, we depolarized the cells with 25 mM KCl in the solution used for Ca^2+^ readdition. As expected, this led to further [Ca^2+^]_i_ elevation compared to the restoration of Ca^2+^ alone (cf. Fig. 1d, f). However, filopodia were retracted. These results are discussed below (see “Exocytosis and stimulus-coupled secretion during extracellular Ca^2+^ replenishment” section).

Filopodia are known to exhibit repetitive cycles of extension and retraction (see “Polarity and Ca^2+^ influx” section). The morphology of the filopodia was known from previous studies [36], and we could estimate their lifetime from time-lapse recordings. Length measurements suggested that they extended at a rate of 1.7 µm/min and remained stable for varying lengths of time. The retraction rate was similar, i.e. 1.4 µm/min (Fig. 1e). The lifetime, 8.5 min (S.D. 6.6, number of filopodia = 10). These rates and lifetimes were in the range of those previously reported for human keratinocytes [88] and for other chicken, mouse, and rat cell lines (for review, see [12, 37]). When compared to filopodia prevalence (cf. Fig. 1a–b, e), the data suggested that approximately two cycles of extension and retraction could occur during the 30-min interval of ER depletion.

The induction of Ca^2+^ influx downstream of receptor-ligand interaction was a hallmark of SOCE [10, 77, 80]. Signaling to the IP_3_R could be sustained by providing EGF during the first part of the Ca^2+^ depletion-readdition protocol (see “Changes in filopodia prevalence during the two-step procedure” section). In order to induce filopodia extension, a duration of 30 min of CPA treatment was needed. Thus, when cells were subjected to depletion for only 20 min before CPA washout and Ca^2+^ restoration, there was little effect (Additional file 1: Fig. 3D). With EGF present in the CPA solution, the effect of Ca^2+^ readdition resembled that observed with the longer, 30-min ER depletion time interval (cf. Fig. 1a,b and Additional file 1: Fig. 3E). This suggested that STIM was activated faster because signaling was the driving force for SOCE. The same argument was applied to determine whether ryanodine receptors enhanced Ca^2+^ release and elevated [Ca^2+^]_i_. The possible role of these receptors was tested by supplying CPA together with the ryanodine receptor agonist, caffeine. We expected that, as for the EGF receptors, the activation of ryanodine receptors would add to the depletion of ER. Caffeine was able to open such channels in the ER store and allow leakage into the cytoplasm. Upon Ca^2+^ readdition, there was little difference in the fraction of cells with filopodia, and coverage of the cell edge with filopodia was reduced in the caffeine-treated cells (Additional file 1: Fig. 3F), which was opposite of the expected effect. This suggested that ryanodine receptors had little role in ER depletion or filopodia formation.

#### Ca^2+^/calmodulin inhibitor, CALP2, and dynasore rescue filopodia during ER depletion

**Fig. 2:**
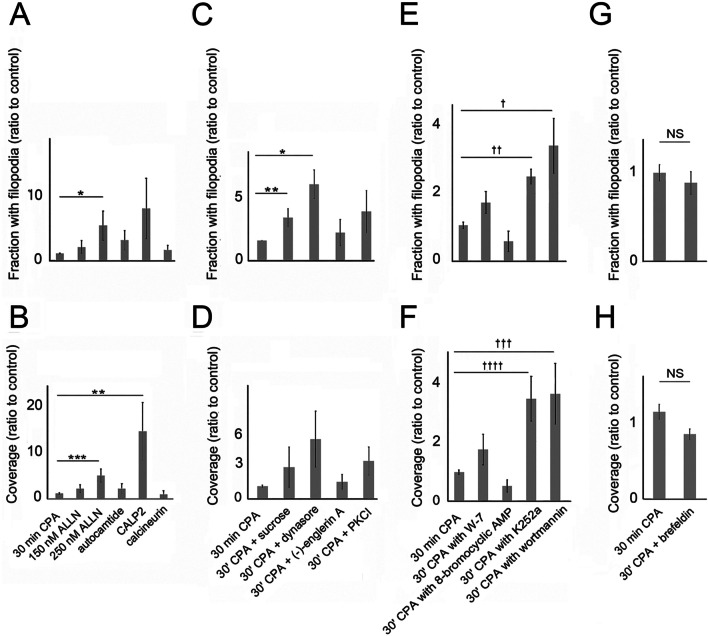
Ca^2+^/calmodulin inhibitor and endocytic inhibitors rescue filopodia during ER depletion. Cells were exposed to 5 µM CPA in Ca^2+^-free HBSS to deplete the ER of Ca^2+^. Inhibitors were used at final concentrations of 1–3 times the IC50 for the targeted activity. Means of 3–7 experiments are shown ± S.E.M. **a, b** Filopodia prevalence in the presence or absence of an inhibitor of calpain (ALLN), CaMKII (10 µM autocamtide-2 related inhibitory peptide), Ca^2+^/calmodulin (20 µM CALP2), or calcineurin (20 µM calcineurin-autoinhibitory peptide II). **a** Significance by ANOVA, *P* = 0.035. *Treatments differ at *P* = 0.037. **b** Significance by ANOVA, *P* = 0.0089. **P = 0.038. ***P = 0.0045. **c, d** Filopodia prevalence in the presence or absence of 0.4 M sucrose, 48 µM dynasore, 23 nM (-)-englerin, or 24 µM protein kinase C inhibitor (PKCI). **c** Significance by ANOVA, *P* = 0.0023. *Treatments differ at *P* = 0.0032. **P = 0.041. **d** Significance by ANOVA, *P* = 0.0032. (E, F) Filopodia prevalence in the presence or absence of W-7 (60 µM), 8-bromocyclic AMP (80 µM), K252a (50 nM), or wortmannin (1 µM). **e** Significance by ANOVA, *P* = 0.00001. ^†^Treatments differ at *P* = 0.0032. ^††^*P* = 0.0004. **f** Significance by ANOVA, *P* = 0.00001. ^†††^Treatments differ at *P* = 0.0056. ^††††^*P* = 0.0012. **g, h** Filopodia prevalence in the presence or absence of 20 µg/ml brefeldin A

The above data showed that ER depletion caused an increase in [Ca^2+^]_i_, that ultimately inhibited filopodia formation. Some calcium-activated proteins previously implicated in filopodial dynamics might have been responsible for the decrease. Previous work on the growth cone had implicated calpain, as well as the Ca^2+^/calmodulin-activated enzymes, calcineurin and CaMKII, in filopodia dynamics [48, 105]. Their role was tested by treating cells with inhibitors of CaMKII, calcineurin, and calpain, as well as Ca^2+^/calmodulin antagonist, CALP2. Calpain inhibitor, ALLN, and Ca^2+^/calmodulin antagonist, CALP2, were the only agents that rescued filopodia (Fig. 2a, b). The effect of ALLN was opposite to that reported for the growth cone, where it had destabilized filopodia and decreased their average lifetime [83]. While 250 µM ALLN was required to block net retraction, this caused cells to round up and most of the cell edge could not be analyzed. Thus, the role of calpain was not investigated further.

It was unlikely that CALP2 affected [Ca^2+^]_i_ directly, because it was previously found to have little effect on [Ca^2+^]_i_ in airway epithelial cells [103]. As CALP2 prevented the retraction part of the filopodia cycle, the result suggested that Ca^2+^/calmodulin increased due to increasing [Ca^2+^]_i_ and affected a downstream target. In neurons, calmodulin is a Ca^2+^ sensor and regulates endocytosis (see for review [117]). We tested the possibility that CALP2 was inhibiting endocytosis by treating cells with dynasore, a known inhibitor of endocytosis. Filopodia were also enhanced by other inhibitors of clathrin-mediated endocytosis, i.e. hypertonic sucrose, a myristoylated pseudosubstrate sequence of protein kinase C (PKC) α/β, PKCI (Fig. 2c, d), and 1-butanol (data not shown).

There is no known mechanism by which Ca^2+^/calmodulin affected endocytosis, but the ubiquitous downstream targets, phosphodiesterase and MLCK, were tested to determine whether they regulated filopodial prevalence. CALP2 was expected to block phosphodiesterase activation by Ca^2+^/calmodulin, with the expected effect being accumulation of cAMP. However, the effects of cAMP analogue, 8-bromocyclic AMP, were opposite of the expected effect (cf. Fig. 2a, b, e, f) and were replicated by forskolin treatment (data not shown). MLCK had been implicated in actomyosin contraction downstream of increases in Ca^2+^/calmodulin. However, MLCK inhibitors, altenusin, MLCK peptide, and ML-7, had little effect over CPA alone (data not shown). This suggested that, despite its effects during long-term exposure, MLCK was ineffective over a 30-min treatment time. K252a, a broad-spectrum kinase inhibitor that inhibits both MLCK and cAMP-dependent protein kinase (PKA) at nanomolar concentrations, rescued filopodia (Fig. 2e, f). This could not be attributed to MLCK. To be consistent with previous reports on protrusion and TRPC5 display on the cell surface [39, 95, 108], cells would have had to retract filopodia after K252a. However, K252a had the opposite effect. As its effect was also opposite of 8-bromocyclic cAMP, the data were consistent with the inhibition of protein kinase A. Not only would this account for the observed result, but K252a was also a PKC inhibitor. Thus, K252a probably replicated the effect of PKCI on PKC, in addition to inhibiting cAMP production (cf. Fig. 2c, d, e, f). While cAMP was produced in response to Ca^2+^ influx in studies of growth cone turning (see for review [6]), treatment of 1000 W cells with 8-bromo-cAMP during Ca^2+^ readdition proved inhibitory (data not shown).

While testing for target proteins affected by calmodulin, we considered the inhibitory effect called Ca^2+^-dependent inactivation (CDI), which is well-documented (see Additional file 1: Additional Results, Targets of Ca^2+^/calmodulin). It should be noted that TRPC1 may not be capable of forming homomeric channels [119], (see for review [7]), but (-)-englerin activated TRPC4 and TRPC5 channels which form heteromeric complexes with TRPC1 [85]. When we treated cells with (-)-englerin to determine whether activating influx through these channels could rescue filopodia, it had little effect (Fig. 2c, d). CALP2 was designed to bind the EF-hands of calmodulin and could mimic the effect of Ca^2+^ in some experiments [103],^104^. To determine whether CALP2 was acting as an agonist or antagonist in the current experiments, we tested the effect of calmodulin inhibitor, W-7. Although it replicated the direction of the CALP2 effect, its effect was weaker (cf. Fig. 2a–d). CALP2 may be binding to more than one of the numerous proteins containing four EF-hands [124]. Lastly, to determine whether CALP2 and dynasore affected filopodia through similar mechanisms, we treated cells with both agents. The effects were no different from CALP2 alone (Additional file 1: Fig. 4A), suggesting that the same mechanism was affected.

#### The CALP2 effect is replicated by inhibiting enzymatically active PI3-K

There are hundreds of calmodulin-binding proteins that might have been affected by CALP2, and its role in regulating filopodia dynamics might depend on multiple protein targets. The above results indicated that dynasore had a similar effect on filopodia. Therefore, we investigated the possibility that CALP2 could affect another Ca^2+^/calmodulin-activated enzyme that regulates endocytosis, namely PI3-K. Ca^2+^/calmodulin binding to the regulatory p85 subunit of PI3-K releases the enzymatically active p110 subunit. Therefore, CALP2 would maintain the catalytic subunit in its inactive form. As this would be duplicated by inhibiting p110, we compared the effect of wortmannin to that of CALP2 (cf. Fig. 2a, b, e, f). Although there are several pathways that are inhibited by wortmannin (see Additional file 1: Fig. 4B), it mainly affects the regulation of vesicle trafficking by PI3-K classes I and II. Inhibiting class I PI3-Ks may decrease the production of phosphatidylinositol 3,4-bisphosphate (PI(3,4)P2) and inhibit endosome maturation. The similarity between the effects of wortmannin and dynasore suggested that the inhibition of filopodia during ER depletion could be reversed by inhibiting endocytic activity.

Wortmannin was known to inhibit both PI(3,4,5)P3 and PI(3,4)P2 synthesis, and so it was expected to affect several stages of endosome formation. Because endocytic compartments are also recycled to make up the membrane of exocytotic vesicles, the effect on filopodia may be indirect. To investigate whether the balance of endocytosis to exocytosis was implicated, we blocked constitutive exocytosis with brefeldin A. This was expected to decrease the membrane pool available for filopodia formation but had no effect over CPA alone (Fig. 2g, h). The effects were negligible, indicating that the supply of membrane available was sufficient to sustain filopodia extension. Rather, the similarity of the inhibitors’ effects (dynasore, hypertonic sucrose, 1-butanol) to that of wortmannin suggested that retraction during ER depletion was due to endocytosis.

#### Sites where STIM1 and TRPC1 coincide with other proteins in untreated cells

**Fig. 3:**
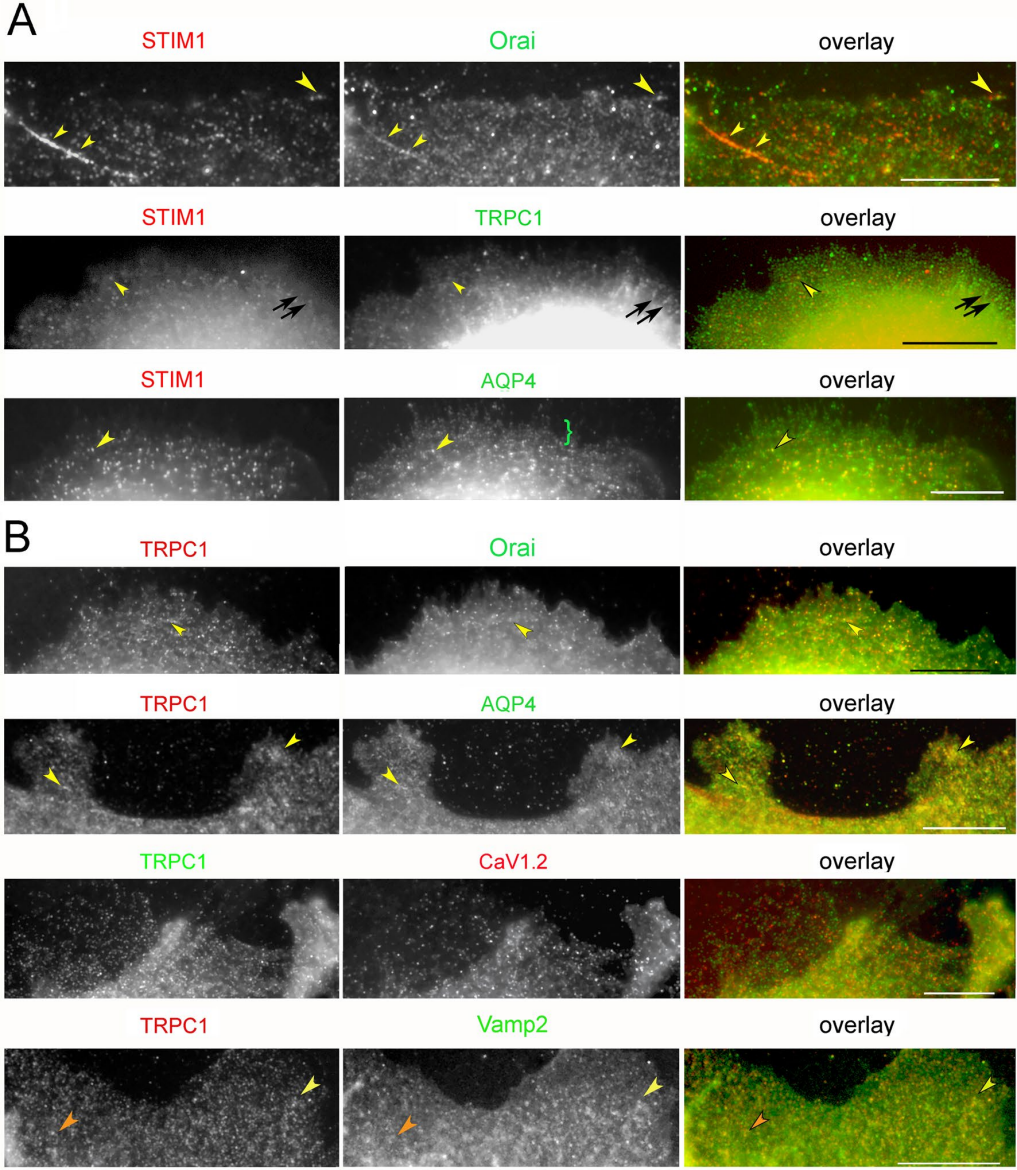
Sites where STIM1 and TRPC1 coincide with other proteins in untreated cells. Loci with colocalized proteins are indicated by yellow arrowheads. Coincidence of the labels in higher parts of the cell was ignored because of the addition of fluorescent signals originating from deeper levels in the cell. **a** STIM1 shows little tendency to colocalize with Orai1 or TRPC1 but consistent colocalization with AQP4. TRPC1 is found in linear arrays (black arrows). AQP4 appears in loci of two distinct size classes, with the smaller confined to the cell edge (green bracket). **b** TRPC1-containing loci contain Orai1 and AQP4 but infrequently the VACC channel subunit, CaV1.2. Areas where the red and green images coincide are mainly in thick portions of the cell where loci are superimposed due to overlapping layers of structure. TRPC1 coincides with Vamp2 in some sites (yellow arrowheads), but in others, diffuse Vamp2 encircles the TRPC1 sites (orange arrowheads). (**b**) TRPC1-containing loci contain Orai1 and AQP4 but infrequently the VACC channel subunit, CaV1.2. Areas where the red and green images coincide are mainly in thick portions of the cell where loci are superimposed due to overlapping layers of structure. TRPC1 coincides with Vamp2 in some sites (yellow arrowheads), but in others, diffuse Vamp2 encircles the TRPC1 sites (orange arrowheads). Bars = 10 µm

Previous work in the field showed that STIM activates Orai channels and binds to TRPC1/4/5 channels (see for review [86]). While untreated cells showed very few sites where STIM1-TRPC1 or STIM1-Orai were colocalized (Fig. 3a, STIM1-Orai and STIM1-TRPC1), STIM1 was commonly colocalized with AQP4. To compare these patterns with a channel unrelated to the SOCE process, we localized AQP4. AQP4 was found together with both STIM1 and TRPC1 in small- and large-sized vesicles (Fig. 3a, STIM1-AQP4 and 3b, TRPC1-AQP4). TRPC1 showed little tendency to occupy vesicles containing the other SOCE mediators. Likewise, colocalization with the channel subunit of the VACC, CaV1.2, and caveolin was rare (Fig. 3b). TRPC5 was known to reside in vesicles that exchange rapidly with the plasma membrane [8], so we determined whether TRPC1 was colocalized with a marker for the rapidly releasable pool (RRP), Vamp2. Similarly, the coincidence of TRPC1 with AQP4 was evaluated by immunofluorescence. Both coincided with TRPC1 more often than other proteins in pairwise combinations with TRPC (Fig. 3b). This was confirmed by determining the Pearson correlations for paired proteins in thin areas of the peripheral cytoplasm. The TRPC1 correlation with AQP4 was significantly higher than with STIM1, Orai, Vamp2, or CaV1.2 (Table 1). Thus, although STIM1 and TRPC1 were rarely found in the same compartment, AQP4 was high in both STIM- and TRPC-containing compartments. This coincided with the bimodal size distribution shown by AQP4 (Fig. 3a, STIM1-AQP4 and 3b, TRPC1-AQP4).

**Table 1:** Correlation coefficients for proteins paired with STIM1 and TRPC1 in untreated cells

Protein coinciding with STIM1		Orai	TRPC1	AQP4
Correlation coefficient		0.509^†^	0.465^‡^	0.816^†,‡^
Standard error		0.043	0.033	0.012
Number of images (N)		11	17	8

Protein coinciding with TRPC1	CaV1.2	Orai	Vamp2	AQP4
Correlation coefficient	0.552^§^	0.520^‖^	0.546^¶^	0.728^§,‖,¶^
Standard error	0.029	0.039	0.030	0.039
Number of images (N)	10	7	8	8

Peripheral cytoplasm from images processed by Colocalization Finder (see Materials and Methods). Significance by one-way ANOVA of comparisons with STIM1 is *P* < 0.00001. ANOVA with TRPC1 is *P* = 0.00031

^†^differ by *P* = 0.00000

^‡^differ by *P* = 0.00000

^§^differ by *P* = 0.00303

^‖^differ by *P* = 0.00046

^¶^differ by *P* = 0.00215

**Fig. 4:**
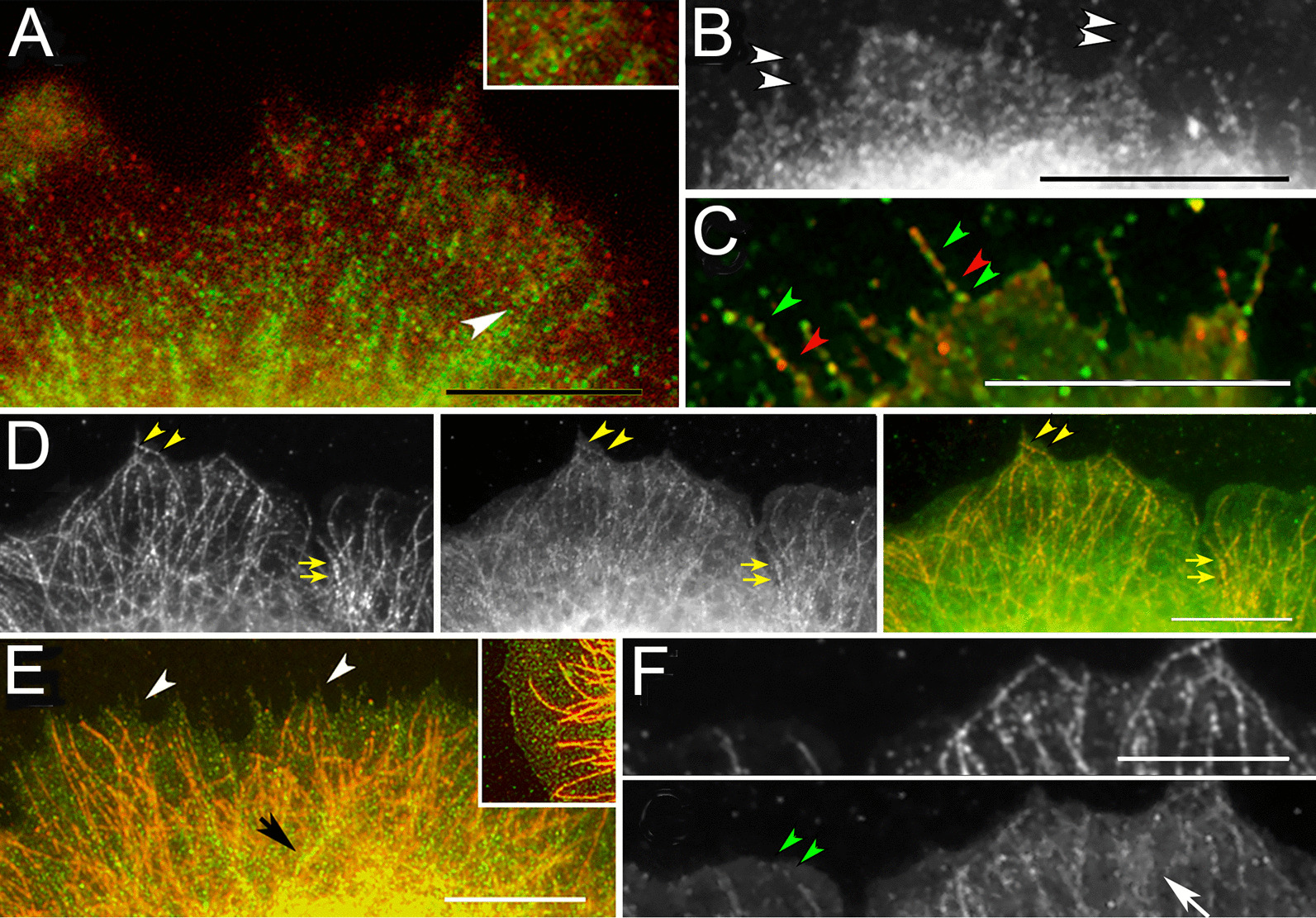
TRPC1 on microtubules and filopodia. **a** Linear arrays of TRPC1 (green) are aligned parallel to features containing STIM1 (red). Inset: enlargement of area designated by arrowhead, showing features side by side. **b** TRPC1 loci aligned on filopodia (white arrowheads). **c** TRPC1 loci (green arrowheads) alternating with STIM1 (red) during Ca^2+^ readdition. **d** Colocalization of β-tubulin (left panel) and TRPC1 (middle panel) in an untreated cell. TRPC1 colocalizes with microtubules bordering the edge (arrowheads) and in the interior (arrows). Panel at right is the overlay. **e** Microtubules (red) labelled by antibody against β-tubulin penetrate to the cell edge but do not enter filopodia (white arrowheads) in an untreated cell. The colocalization of TRPC1 and β-tubulin is apparent in higher parts of the cell (black arrow). Inset: TRPC1 loci (green) free of microtubules (red) at the cell edge. **f** Colocalization of β-tubulin (upper frame) and TRPC1 (lower frame) shows that TRPC1 spots (green arrowheads) extend beyond the ends of microtubules. Interior to the edge, TRPC1 is found in diffuse areas around the microtubules (white arrow). Bars = 10 µm

Although TRPC1 and STIM1 rarely occupied the same compartment in untreated cells, they were found in parallel structures (Fig. 4a). Similarly, on filopodia, TRPC1 occupied alternating sites with STIM1 rather than colocalizing with it (Fig. 4b, c). Colocalization of TRPC1 with β-tubulin demonstrated that TRPC1 was in close proximity to microtubules (Fig. 4d). STIM is trafficked as a microtubule end-tracking protein [32]. Hence, the TRPC1 localization suggested it was also aligned with microtubules. Some of the vesicles were found unattached since, in thin portions of the cell edge, some TRPC1 loci were far from the microtubules (Fig. 4e, f). These data confirmed that most TRPC1 occupied vesicles with little representation of the other components studied, except for AQP4. These observations were supported by data shown in Table 1.

#### Loci containing STIM1, Orai, TRPC1, AQP4, and Vamp2 during ER depletion

There were significant changes in the correlation coefficients of STIM1 or TRPC1 with other proteins during ER depletion. While the TRPC1-Vamp2 correlation increased (Table 2), correlations between TRPC1 and other proteins, caveolin, Orai, and VACC channel alpha-1 subunit, were unchanged (Additional file 1: Table S1). STIM1 and Orai, when localized after ER depletion, coincided in areas containing discrete STIM1 punctae (Additional file 1: Fig. 5A). The formation of STIM1 punctae is nearly universal (see for review [14, 22, 77]). However, a Pearson correlation analysis of STIM1-Orai failed to show a difference after ER depletion. This was surprising, but upon further investigation, we found that the STIM-Orai puncta occurred at sites internal to the cell edge (cyan areas in Additional file 1: Fig. 5I). As these areas were rarely included in the images used for correlation analysis (see “Correlation coefficients” section), they contributed little to the correlation values. Although they were as large as the punctae formed after overexpression, they were present in only a fraction of cells sampled at 30 min (see “Characteristics of the SOCE mediators and other proteins after Ca^2+^ replenishment” section).

**Table 2:** Correlation coefficients for proteins colocalized with TRPC1 before and during SOCE

Experimental phase	Untreated	ER depletion	Ca^2+^ replenishment
*AQP4*			
Correlation coefficient	0.727^†,‡^	0.845^†^	0.910^‡^
Standard error	0.039	0.019	0.020
	8	6	8

*STIM1*			
Correlation coefficient	0.465^§^	0.490^‖^	0.786^§,‖^
Standard error	0.033	0.029	0.026
Number of images	17	6	7

*Vamp2*			
Correlation coefficient	0.546^¶,#^	0.714^¶^	0.791^#^
Standard error	0.033	0.054	0.040
Number of images	8	6	7

Peripheral cytoplasm from images processed by Colocalization Finder (see “Immunofluorescence localization, image acquisition, and image processing” section)

^†^ANOVA on experiment, *P* = 0.00037, treatments differ at *P* = 0.0188

^‡^Treatments differ at *P* = 0.00049

^§^ANOVA on experiment, *P* < 0.00001, treatments differ at *P* = 0.00001

^‖^Treatments differ at *P* = 0.00002

^¶^ANOVA on experiment, *P* = 0.00047, treatments differ at *P* = 0.014

^#^treatments differ at *P* = 0.00061

**Fig. 5:**
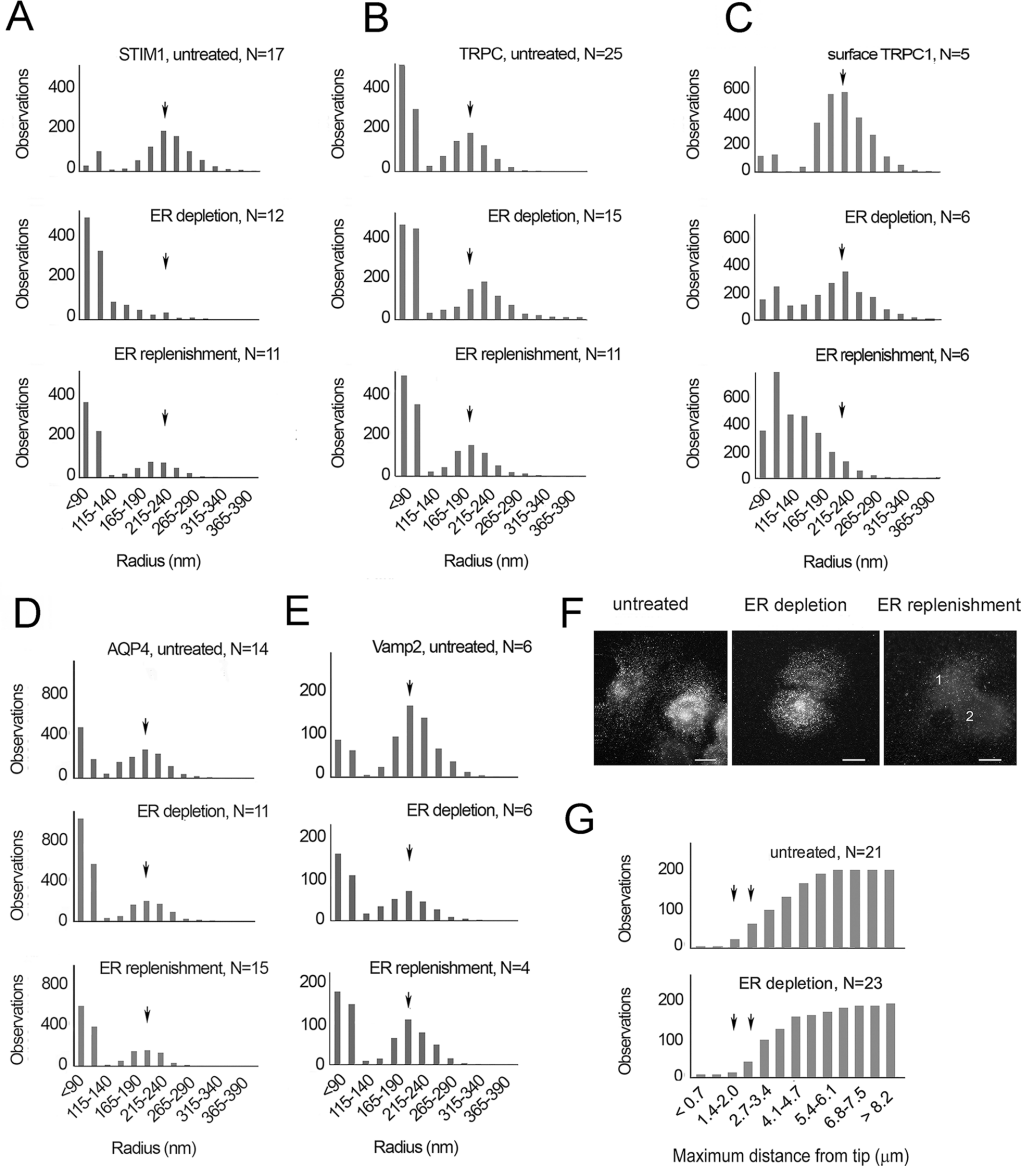
Loci containing STIM1, Orai, TRPC1, AQP4, and Vamp2. Results represent 3–8 experiments. **a–f** The peak radius for each vesicle size distribution in the untreated cell sample is indicated by an arrow and the same mark applied to samples collected during ER depletion and Ca^2+^ readdition. **a** STIM1 peak at 240 nm, **b** TRPC1 peak at 200 nm, **c** cell surface TRPC1 peak at 215 nm, **d** AQP4 peak at 205 nm. **e** Vamp2 peak at 205 nm, **f** cells were fixed but not permeabilized after no treatment (untreated), after Ca^2+^ depletion, or after Ca^2+^ depletion followed by Ca^2+^ readdition. TRPC1 shows a diffuse localization in cells 1 and 2. **g** Cumulative distribution of TRPC1 loci in samples of untreated cells and cells after ER depletion. The circumference within which loci are found near each filopodium is represented. Loci within 2.7 µm of the filopodia tip are slightly fewer during ER depletion. Statistics on the distances are untreated (mean = 3.27, S.E.M. 0.091 µm, 196 loci) and ER depletion (mean = 3.78, S.E.M. 0.13 µm, 187 loci). The number of particles segmented was closely related to the number of images (N) analyzed, and N was linear with the number of particles recovered within a single treatment (see Additional file 1: Fig. 6A–B)

To gain further insight, the loci containing SOCE mediators were analyzed quantitatively by modeling them as particles. Those with a circular shape were segmented (see “Analysis of particle sizes after staining by indirect immunocytochemistry” section). When the size distributions were plotted for STIM1, the peak size differed dramatically in CPA-treated samples compared to controls (Fig. 5a). Numerous loci of 140 nm or less were thought to represent the well-known punctae in the plasma membrane [4, 14, 32, 56]. The peak radius of TRPC1 loci changed in the opposite direction (Fig. 5b), from ~ 200 to ~ 220 nm, and this change was reflected in a slight shift to more internal positions in the cytoplasm.

In addition to vesicular sites, TRPC1 occupied loci on the cell surface. When these sites were identified by localizing the extracellular portion of the TRPC1 channel (see “Immunofluorescence localization, image acquisition, and image processing” section), their peak remained the same before and during ER depletion (Fig. 5c). As the loci immunodecorated with anti-AQP4 or anti-Vamp2 were unchanged, only TRPC1 and STIM1 trafficking showed evidence of being affected by ER depletion (Fig. 5a–e). The apparent distribution of TRPC spots on the cell surface remained the same until Ca^2+^ readdition (Fig. 5f). To acquire quantitative data about their spatial distribution, we mapped TRPC loci in the regions around filopodia. This was done by localizing TRPC1, then counting the clusters within 8 µm of each filopodium. TRPC1 representation was reduced in these areas after ER depletion (Fig. 5g). As the filopodia prevalence did not change (Fig. 1a, b), this suggested a lowered density of surface TRPC1 during ER depletion.

**Fig. 6:**
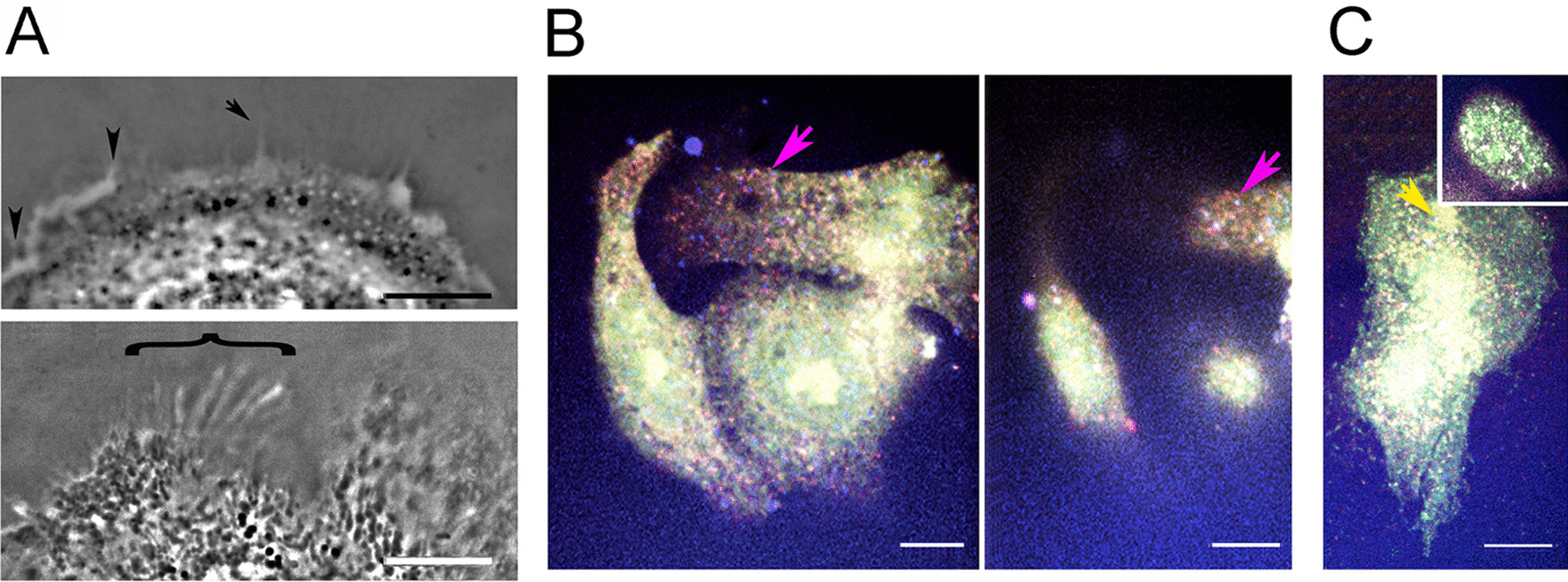
Functional aquaporins and STIM1-labelled loci in relation to SOCE channels. **a** Areas with low refractive index are indicated by lower phase density. (Top) Low density areas are found immediately under the plasma membrane (arrowhead), in vesicles near the cell edge, in elevated portions of the cell, and in pointed protrusions (arrow). (Bottom) Linear structures with low density are present in the lamellipodium (bracket). **b, c** Confocal planes from cells after Ca^2+^ readdition and colocalization with antibodies against STIM1 (blue), Orai (green), and TRPC1 (red). (**b**, Left) Ventral plane with diffuse Orai (green) and numerous areas of STIM-TRPC colocalization in magenta (arrow). (Right) Dorsal plane of the same cells, **c** ventral plane of another area showing Orai colocalized with TRPC1 (yellow arrowhead) and areas of STIM1-TRPC1 colocalization (magenta loci). Inset: Nucleus of the same cell showing the diffuse distribution of Orai (green) and colocalization of all three proteins (white). Bars = 10 µm

When the correlation coefficients between AQP4 and TRPC1 in peripheral areas were determined, their values increased significantly during ER depletion (Table 2). Since aquaporins are also implicated in polarity determination, they could serve as a basis for comparison with the SOCE mediators. To determine where aquaporins were effective in polarity, we visualized areas showing a lowered refractive index in the cells. These included ruffles, rounded and pointed protrusions, and vesicles, suggesting that aquaporin channels were active both in the cell interior and at the plasma membrane (Fig. 6a).

#### TRPC1 dissemination in the plasma membrane in CALP2- or dynasore-treated cells

Some of the TRPC1 molecules were disseminated in the plasma membrane after Ca^2+^ readdition (see “Characteristics of the SOCE mediators and other proteins after Ca^2+^ replenishment” section). As this accompanied filopodia formation, we explored the possibility that dynasore or CALP2 caused dissemination during ER depletion. Comparing CALP2- or dynasore-treated cells with those treated by ER depletion alone, we found a greater representation of external loci with a radius < 140 nm (Additional file 1: Fig. 6A–B). The results were unlikely to be caused by increased exocytosis of TRPC1, as Ca^2+^/calmodulin rarely played a role in exocytosis. One well-known exception was an activation mechanism mediated by CaMKII. This caused phosphorylation of synapsins and release of synaptic vesicles [38]. The CaMKII inhibitor, autocamtide-2 related inhibitory peptide, had no effect during the first step of the protocol, so this could not be the mechanism of the CALP2 effect (Fig. 2a–b). Thus, the dissemination by CALP2 and dynasore may have caused by trapping TRPC1 in the plasma membrane. Immunochemical localizations conducted in untreated cells showed that most TRPC1 resides in intracellular vesicles. If these represented a recycling compartment, TRPC and other proteins in the compartment could be released onto the cell surface and trapped there by CALP2 or dynasore treatment.

### Characteristics of the SOCE mediators and other proteins after Ca^2+^ replenishment

It is well established that, after Ca^2+^ readdition, STIM punctae detach from sites at the cell surface and return to the ER (see for review [77]). To determine how the timing of this process compared to filopodia formation, we localized the SOCE mediators after Ca^2+^ readdition. While present in a minority of cells, Orai-STIM1 punctae were still observed and resembled those seen during ER depletion (cf. Additional file 1: Fig. 5A–B). Diffuse sites of TRPC1-STIM1 colocalization appeared on the plasma membrane (Additional file 1: Fig. 5C). Obvious Vamp2-TRPC1 colocalization also occurred on the plasma membrane, and Vamp2 appeared to be secreted like TRPC1. The Pearson correlations for Vamp2-TRPC1 image pairs indicated a significant increase in colocalization over untreated cells (Table 2). This was consistent with other data indicating that Vamp2 and TRPC1 resided in the RRP, albeit in separate vesicle subtypes (see (Additional file 1: Fig. 5D–E). In contrast, sites localized by antibodies against caveolin and the voltage-activated channel subunit, CaV1.2, showed no change during ER depletion or after Ca^2+^ readdition. Those protein pairs whose correlations with TRPC1 remained unchanged during the two-step procedure are listed with their coefficients in Additional file 1: Additional Results (Table 1). Other laboratories observed integrin β1 on filopodia [68]. As voltage- and Ca^2+^-activated K^+^ channels, called BK channels, were regulated by Src-mediated phosphorylation downstream of integrin β1 (see for review [37]), we wanted to determine whether integrin was related to SOCE-initiated changes. It was typically found in diffuse distributions or attached to cytoskeletal structures (Additional file 1: Fig. 5F). Integrin-TRPC1 correlations resembled those of caveolin or CaV1.2 and were unaffected by SOCE (data not shown).

Aquaporins were further investigated because, like the SOCE mediators, they were linked to polarity determination [46], (see for review [75, 92]). By conducting water into the space underneath the plasma membrane, the aquaporins can increase the rate of actin polymerization [45], (see for review [59]), which may facilitate filopodia extension. The correlation coefficients of TRPC1-AQP4 increased after ER depletion and remained high after Ca^2+^ readdition (Table 2). Although it is possible that it facilitated filopodia extension, aquaporin secretion alone could not explain all the current findings.

To determine whether all three proteins mediating SOCE coincided, STIM1 (blue), Orai (green), and TRPC1 (red) were colocalized. Despite data suggesting that TRPC1 and STIM1 were trafficked in antiparallel directions after Ca^2+^ readdition (see below), the two molecules were often colocalized (Fig. 6b, magenta arrows). It was noteworthy, however, that both TRPC1 (red) and STIM1 (blue) were found at the cell edge. STIM1-Orai coincidence was less frequent (cf. Fig. 6b (cyan) and Additional file 1: Fig. 5I). Whereas TRPC1-Orai coincidence was rare, some sites were observed in locations internal to the edge (cf. Fig. 6c and Additional file 1: Fig. 5G–I (yellow arrowheads). All three proteins were found to be colocalized in upper portions of the cell (Fig. 6c, white areas).

After Ca^2+^ was restored, the size distribution of STIM1 loci reverted toward that of untreated cells (Fig. 5a), suggesting that STIM began to return to its original residence in the ER. The peak value of internal TRPC1 loci had shifted to slightly greater sizes during ER depletion, but after Ca^2+^ readdition, it also reverted toward its original value (Fig. 5b). In contrast, under conditions where the extracellular sites were detected (see “Immunofluorescence localization, image acquisition, and image processing” section), the peak size of TRPC1 loci was drastically reduced. The peak of this distribution was 105 nm, corresponding to the diffuse localization of TRPC1 in images (cf. Fig. 5c, f). These data indicated that TRPC1 was secreted onto the plasma membrane. Altogether, the results suggested that STIM1 and TRPC1 molecules occupied separate compartments in untreated cells, and their trafficking was in opposite directions during the two steps of SOCE induction. Comparing these data with the levels of cytoplasmic Ca^2+^, which approached those of untreated cells by 15 min after Ca^2+^ readdition (Fig. 1d), we concluded that STIM1 and TRPC1 molecules shared the same compartment following Ca^2+^ readdition, i.e. while [Ca^2+^]_i_ was high and falling. This conclusion was consistent with the paired proteins’ correlation (Table 2).

### Filopodia formed during Ca^2+^ replenishment depend on TRPC1/4/5

**Fig. 7:**
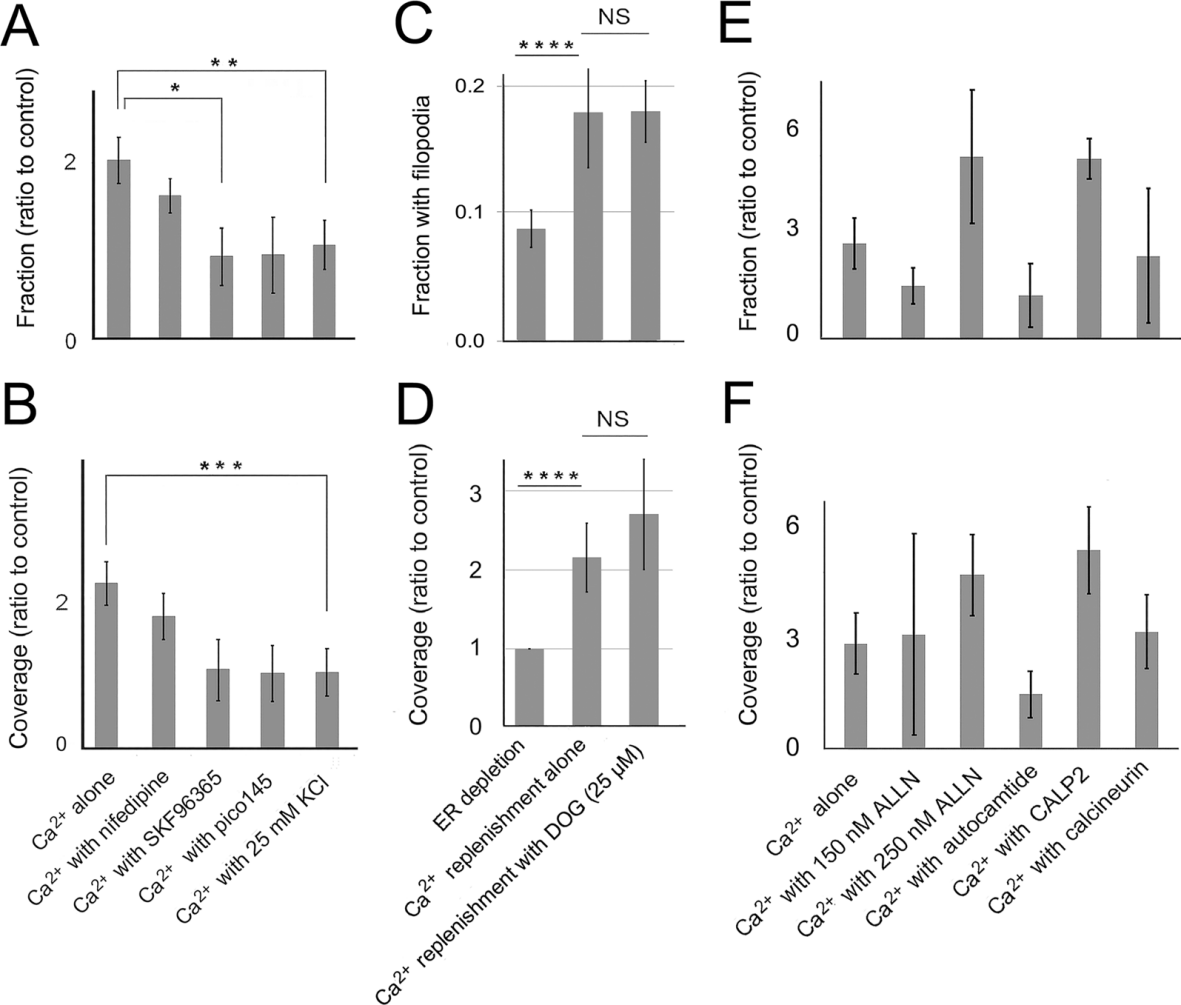
Ca^2+^ channels and effectors implicated in net filopodia formation. **a–f** Cells in Ca^2+^-free HBSS were treated with CPA, rinsed, and treated in the second phase by readdition of the extracellular Ca^2+^ prior to fixation. Results are representative of 3–9 experiments. **a, b** Filopodia prevalence after Ca^2+^ readdition alone (Ca^2+^ alone) or with 8 µM nifedipine, 10 µM SKF96365, 15 nM pico145, or 25 mM KCl. **a** Significance by ANOVA, *P* = 0.0075, *P = 0.032, **P = 0.047. **b** Significance by ANOVA, *P* = 0.012, ***P = 0.027. **c, d** Filopodia prevalence of cells treated with readdition alone (Ca^2+^ alone) or with 25 µM dioctylglycerol (DOG), **c** significance by ANOVA *P* = 0.0056, ****P = 0.011. **d** Signficance by ANOVA 0.00075, ****P = 0.020. **e, f** Filopodia prevalence of cells treated with readdtion alone (Ca^2+^ alone), ALLN, 10 µM autocamtide-2 inhibitory peptide, 20 µM CALP2, or 20 µM calcineurin inhibitory peptide. Significance by ANOVA, *P* = 0.165

As the above data showed that both internal TRPC1 loci and surface clusters were disseminated after Ca^2+^ readdition, surface TRP appeared important for filopodia extension. A number of channel inhibitors were tested to determine whether any channel activity significantly affected extension. SKF96365, as well as a specific antagonist of TRPC1, 4, and 5 heteromeric complexes, pico145 [84], inhibited filopodia. Nifedipine, a VACC inhibitor, had a slight inhibitory effect (Fig. 7a, b). To determine whether the subfamily of TRPC3/6/7 isoforms contributed to filopodia extension, Ca^2+^ was restored in the presence or absence of dioctylglycerol (DOG). This had no effect (Fig. 7c, d), and so the TRPC3/6/7 subfamily did not appear to facilitate filopodia formation.

### Exocytosis and stimulus-coupled secretion during extracellular Ca^2+^ replenishment

**Fig. 8:**
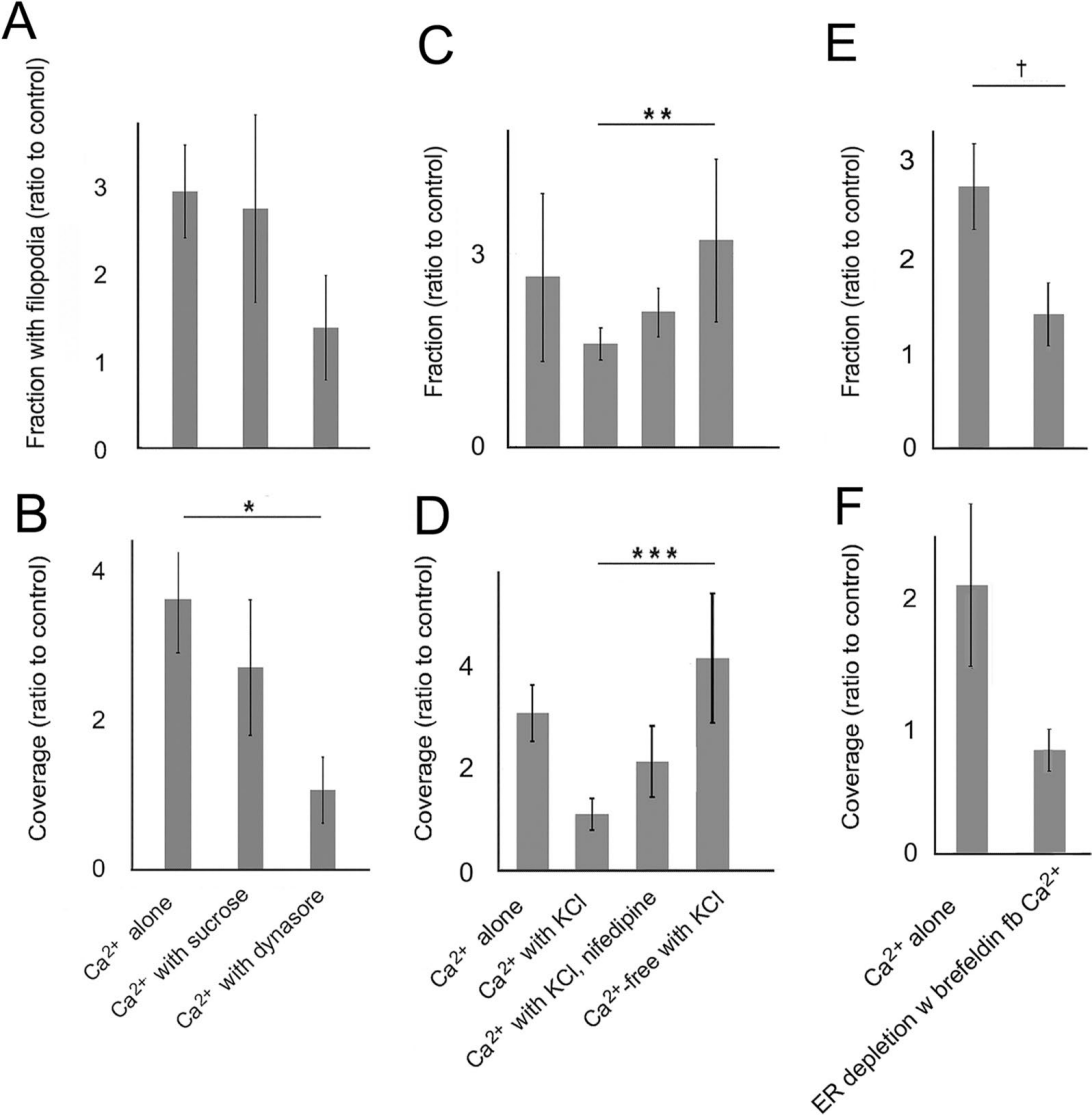
Effects of endocytic and exocytotic inhibitors on filopodia prevalence after Ca^2+^ readdition. Results shown are representative of 4–9 experiments. **a**–**d** Filopodia prevalence after Ca^2+^ alone (Ca^2+^ alone) or in the presence of various inhibitors, **a, b** Ca^2+^ readdition in the presence or absence of 0.4 M sucrose or 48 µM dynasore. **a** Significance by ANOVA, *P* = 0.153, **b** significance by ANOVA, *P* = 0.0034, *P = 0.038. **c, d** Ca^2+^ readdition in the presence or absence of 25 mM KCl with and without nifedipine or 25 mM KCl in Ca^2+^-free HBSS (Ca^2+^-free). **c** Significance by ANOVA, *P* = 0.049, **P = 0.033. **d** Significance by ANOVA, *P* = 0.022, ***P = 0.012. **e, f** Samples pretreated during ER depletion with or without brefeldin (20 µM) followed by Ca^2+^ readdition. **e** ^†^Treatments differ at *P* = 0.044

The above evidence showed that Ca^2+^ readdition initiated secretion of TRPC1 molecules, from vesicles that contained some Vamp2 (cf. Fig. 5f, Additional file 1: Fig. 5C, Table 2). Therefore, we anticipated that inhibitors of endocytosis would cause even greater TRPC1 accumulation. To test this, along with the possibility of calcineurin or CaMKII participation, we determined the effects of all of the inhibitors used during ER depletion. Now, neither ALLN nor CALP2 had a statistically significant effect (cf. Figs. 2a, b and 7e, f). Surprisingly, the endocytic inhibitors, hypertonic sucrose and dynasore, now decreased filopodia prevalence (cf. Figs. 2c, d and 8a,b). One possible explanation was that Ca^2+^ readdition allowed TRPC to be recycled rapidly. Data obtained with Pico145 and SKF96365 showed that TRPC1, 4, and/or 5 channels must be activated to increase filopodia prevalence. If inhibitors of endocytosis blocked the channels’ reentry and caused a failure of recycling, it would imply that TRP molecules or proteins associated with them must be recycled through the RRP. On the other hand, one target of dynasore, the GTPase dynamin-2, has been implicated in fusion pore regulation for exocytosis [29]. Because sucrose also inhibited filopodia, i.e. an effect similar to dynasore, the data suggest that it was recycling and not merely exocytosis that is required for filopodia formation after Ca^2+^ readdition.

We proposed that the application of endocytic inhibitors during ER depletion had caused TRPC1 to be trapped at the cell surface (see “TRPC1 dissemination in the plasma membrane in CALP2- or dynasore-treated cells” section). We further explored the question of how the agents showed the opposite effect, i.e. inhibiting filopodia, after Ca^2+^ readdition. In the presynaptic nerve terminal, the rate of RRP replenishment from a reserve pool of vesicles depended on Ca^2+^/calmodulin [118]. If the levels of Ca^2+^/calmodulin increased during ER depletion, it would support an increased volume of the downstream RRP and the availability of greater numbers of RRP vesicles upon Ca^2+^ readdition. In endocrine cells, secretory vesicles were coupled to the stimulus of Ca^2+^ influx through VACCs (see for review [65, 93]). As [Ca^2+^]_i_ was increased when 25 mM KCl was included in Ca^2+^-replete medium (Fig. 1f), the data supported other evidence showing that VACCs existed in airway epithelial cells in situ [103]. We hypothesized that the stimulus-coupled activity might increase filopodia prevalence over the levels obtained with Ca^2+^ readdition alone. Contrary to expectations, this was inhibitory (Fig. 7a, b). The simplest explanation for the net retraction was Ca^2+^-dependent inhibition of Orai or TRPC1/4/5 (see Additional file 1: Additional Results, Targets of Ca^2+^/calmodulin).

Because activated STIM1 is known to inhibit L-type VACCs, either by retaining them in internal compartments [76] or recruiting them into punctae [114], the ability of VACCs to open was expected to be moderated during ER depletion. As the results of Fig. 1f showed that VACCs must have been exposed at the cell surface, any STIM-mediated inhibitor effect must have been counteracted by depolarization. To determine whether Ca^2+^ influx was itself the target of depolarization, cells were treated with KCl and the classic inhibitor of VACCs, nifedipine. This slightly reduced the effect of KCl, reaffirming that Ca^2+^ influx was inhibitory. However, a much greater effect was observed when KCl was delivered in Ca^2+^-free media (Fig. 8c, d). As only internal Ca^2+^ was available under these conditions, this confirmed that Ca^2+^ influx was not strictly required for filopodia extension. An active TRPC channel was required, however, as noted above.

If TRP exocytosis was required for filopodia extension, we reasoned that it could be constrained by reducing the reserve pool. By blocking constitutive membrane transport from the ER to this compartment with brefeldin A during ER depletion, we could inhibit filopodia formation after Ca^2+^ readdition (Fig. 8e, f). In contrast, during ER depletion, this pool had played no role in rescuing filopodia (see “The CALP2 effect is replicated by inhibiting enzymatically active PI3-K” section). This confirmed the importance of a downstream exocytotic compartment in filopodia extension.

### Anchorage in the signalplex and cytoskeleton

**Fig. 9:**
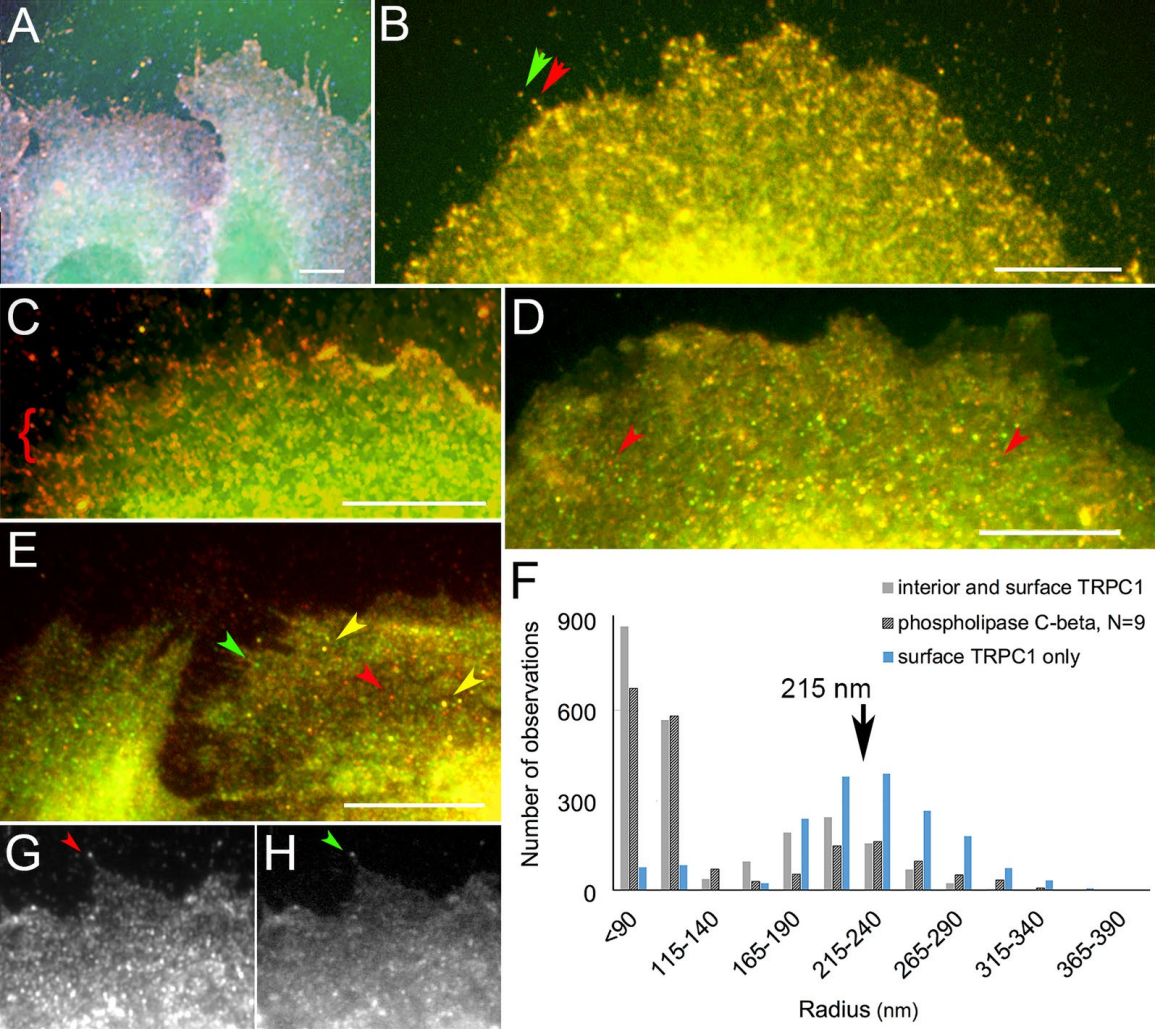
Colocalization of PLC isoforms and TRPC1. **a** Triple localization of PLCβ2 (green), PLCγ1 (blue), and TRPC1 (red). PLCγ1 and TRPC1 coincide in interior parts of the cells (lavender). **b, c** Colocalization of PLCβ2 (green) and TRPC1 (red). **b** In an untreated cell, the labels coincide even at the cell edge (arrows). **c** After Ca^2+^ readdition, TRPC1 is concentrated at the cell edge (bracket) but the two proteins are often colocalized at the interior of the cell (yellow). **d, e** Colocalization of PLCβ2 (red) and PLCγ1 (green). **d** In an untreated cell, the proteins coincide in many sites (yellow), but PLCβ2 can also occupy a separate compartment (arrowheads). **e** After Ca^2+^ readdition, the proteins share compartments in some sites (yellow arrowheads) but not in others (green and red arrowheads). **f** Cumulative distribution of PLCβ2 particle sizes. The peak radius of loci in images from untreated cells is indicated. **g, h** Localization of PLCβ2 (**g**) and PLCγ1 (**h**) at the cell edge, showing a concentration at the pointed feature (arrowhead)

Whereas it is thought that the display of TRP channels is subject to regulation by actin, this is easily explained by the role of actin filaments in endo- and exocytosis. The opposite process has recently been reported, namely the organization of actin around channels [67, 96]. The extreme C-terminal sequence of TRPC4/5 binds to a PDZ motif at the N-terminal end of Na + /H + exchanger regulatory factor 2 (NHERF2). PDZ motifs, named after the postsynaptic density protein, PSD95, are docking sites for other proteins. A C-terminal domain of NHERF then binds an ERM (ezrin, radixin, and moesin) protein. The C-terminal actin-binding domain, which also contains the NHERF-binding site, allows ERM proteins to link to actin filaments (see for review [43]. TRPC clusters may contain phospholipase C (PLC), isoforms β1/2 and γ, which interact with the first or second PDZ domain of NHERF [100, 107]. Thus the whole resembles a *Drosophila* signalplex, which consists of PLC, PKC, TRP, and a scaffold protein in stochiometric concentrations [110]. A signalplex allows proteins to be assembled in a specific order like a daisy chain (see Discussion). To assess this possibility, we colocalized PLCγ, TRPC1, and PLCβ2. The triple localization showed that TRPC1 (red) was present with PLCγ (blue) in interior portions of the cell (Fig. 9a). Sites where PLCβ2 was found with TRPC1 were suggestive of assembly in the surface clusters (Fig. 9b, c). TRPC1-PLCβ2 correlation coefficients averaged 0.74 for untreated samples but ranged as low as 0.46 in samples collected after Ca^2+^ readdition. This would be consistent with the knowledge that TRPC1 is disseminated. PLCβ2-bearing loci varied in their content of PLCγ (Fig. 9d). Spherical particles containing PLCγ appeared less prominent at the cell edge, but the two proteins continued to coincide in some sites after Ca^2+^ readdition (Fig. 9e). Upon determining the size distribution of PLCβ2 loci, we found a peak radius of 215 nm, coinciding with that of surface TRPC1 clusters (Fig. 9f). It was notable that both PLC isoenzymes were found at the tips of filopodia, where they colocalized with TRPC1 (Fig. 9b, g, h). These results suggested that surface clusters containing TRPC1 contained PLCβ2, and that TRPC1 dissociation after Ca^2+^ readdition caused dissolution of some of the sites of TRPC1-PLC coincidence. Although the images of PLCγ loci were not robust enough to allow particle size determination, possibly because the antibodies against PLCγ had low avidity compared to the other antibodies, PLCγ correlations with TRPC1 and PLCβ2 did not appear changed by different phases of SOCE. The results suggest that the activation of PLC by Ca^2+^ influx may cause breakdown of phosphatidylinositol 4,5-bisphosphate (PI(4,5)P2, also called PIP2), which would be consistent with both the signalplex structure and the dissemination of TRPC channnels to form a homogeneous distribution after Ca^2+^ readdition. These mechanisms are discussed further below (see “Discussion” section).

Whereas the results suggest that the process of TRPC1 dissemination was duplicated by treating cells with CALP2 or dynasore during ER depletion, this may not have been caused by the same hypothetical PLC activation. After Ca^2+^ readdition, TRPC1 is thought to be disseminated at least in part by exocytosis of vesicles from the RRP, which is in turn influenced by the size of the reserve pool. The effect during ER depletion was attributed to TRP channel trapping (see “TRPC1 dissemination in the plasma membrane in CALP2- or dynasore-treated cells” section).

## Discussion

Filopodia are the sensors for both chemotactic and haptotactic signals, and they set the direction of motility for whole cells [3, 11, 87], as well as the nerve axon (see “Background” section). The role of Ca^2+^ flux in filopodia dynamics has been puzzling, however. When calcium entry was induced by the opening of voltage-gated calcium channels, it caused filopodial extension. This was followed by retraction despite the continued elevation of intracellular Ca^2+^ [20, 81]. Other studies, in which calcium was released intracellularly by photolysis of caged Ca^2+^ compounds, showed a brief rise in local calcium concentration which was rapidly followed by restoration to the baseline. Filopodia extension occurred when the calcium level was declining [52]. This agreed with data on spontaneous Ca^2+^ transients in dendrites, where low intracellular Ca^2+^ was found to coincide with filopodia formation [58]. In classical studies of growth cone filopodia, Kater and coworkers described a “bell-shaped” relationship between calcium and filopodia extension. Conditions were favorable for filopodia formation when [Ca^2+^]_i_ was either low and rising or high and falling [47]. Whereas these data suggested a consistent pattern, there were other results suggesting that temporal, i.e. frequency, or directional modulation of Ca^2+^ flux were important [83].

In hematopoietic cells, i.e. neutrophils and lymphocytes, which are commonly used model systems for studying SOCE, an agonist typically elevates [Ca^2+^]_i_ within 1–3 min. Then, the level spontaneously declines to baseline [13, 15, 28, 61, 109, 122]. The rate of decline is governed by the rate of reuptake into the ER and the rate of exhaust through the plasma membrane calcium pump [60]. Although epithelial cells are more rarely the subjects of study, we find their kinetics of both [Ca^2+^]_i_ increases and decreases are slower (see [57] and the current article). By chance, the rise in [Ca^2+^]_i_ that occurred in the 1000 W cells was prolonged, as it was in axonal growth cones [20, 81], and this allowed us to replicate the “bell-shaped” pattern.

### TRPC localization and activity follow a bell-shaped pattern

Although there was a requirement for extracellular Ca^2+^ for cells to extend filopodia over a long term [123], the levels of intracellular Ca^2+^ were sufficient to complete one or two cycles of extension and retraction while [Ca^2+^]_i_ was rising. Whereas this confirmed that Ca^2+^ influx was dispensable, it did not show that TRP channels were dispensable, because residual channels in the plasma membrane may have sufficed to maintain one or two cycles. Later, the ability to support filopodia growth was lost. Along with external Ca^2+^, extracellular ligands were removed during ER depletion. In this phase, the normal trafficking of TRPC was interrupted, and it was withdrawn in vesicles into internal compartments. As the pattern of TRPC1 distribution in the plasma membrane was the outcome best correlated with filopodia formation during the rise and fall in [Ca^2+^]_i_, the effect of SOCE induction closely followed the pattern of TRPC channel display. One advantage of using the current experimental design was that the media used for Ca^2+^ readdition was devoid of ligands that activated upstream signaling. Thus, the system allowed the immediate effects of SOCE-generated Ca^2+^ to become more prominent than the upstream mechanisms stimulating IP_3_R.

When considering how the increase in global [Ca^2+^]_i_ and/or lack of Ca^2+^ flux prevented filopodia formation, we had previously rejected a role for CDI. Although it was known that TRPC4 bound Ca^2+^/calmodulin when global Ca^2+^ levels were elevated [112], it was deemed unlikely that Ca^2+^ levels this high were reached under our ER depletion conditions (see Additional file 1: Additional Results, Targets of Ca^2+^/calmodulin). After CPA washout and Ca^2+^ readdition, global [Ca^2+^]_i_ was falling despite the obvious display and activation of TRPC channels. If cell depolarization was induced simultaneously with Ca^2+^ readdition, a rapid rise of Ca^2+^ could be observed due to VACC activation. VACCs reside within laterally confined regions of the plasma membrane, and their activation is expected to elevate local Ca^2+^ to micromolar concentrations. Indeed, at these higher levels, TRP channels disseminated on the cell surface may be closed by CDI (see Additional file 1: Additional Results, Targets of Ca^2+^/calmodulin). Of these two channel classes, the VACCs are more highly selective for Ca^2+^ (see for review [7]). Na^+^, which is in ~ 75-fold excess over divalent cations in Ca^2+^-replete HBSS, is likely to predominate over Ca^2+^ entering through the TRPC channels. Thus, the Ca^2+^ influx through Orai and VACC had vastly different consequences. The data suggested that the small Ca^2+^ influx through Orai is favorable for rapid deployment of TRPC from the RRP, but high Ca^2+^ influx inhibited the channels’ activity.

#### Ca^2+^ readdition stimulates TRPC secretion

**Fig. 10:**
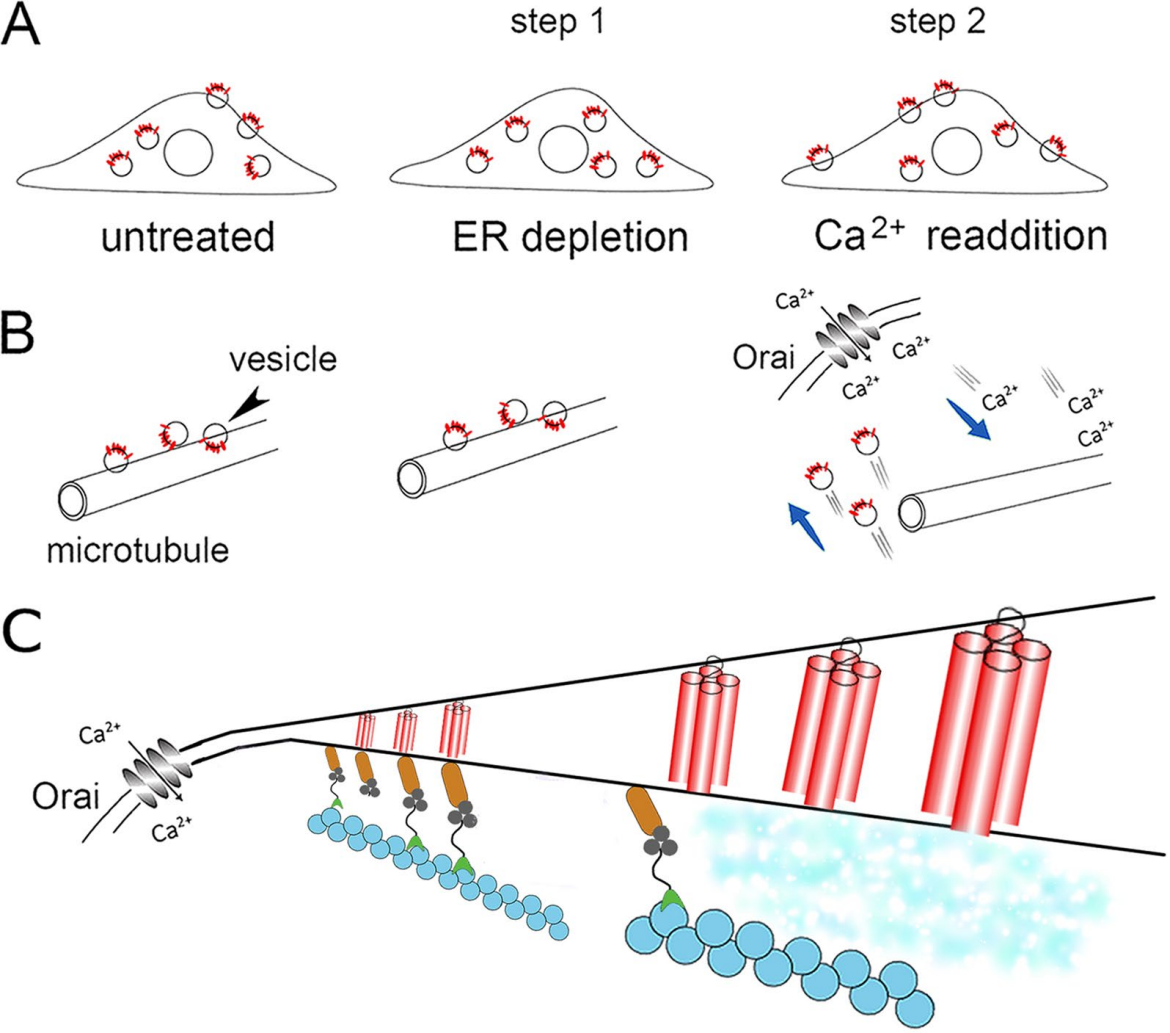
SOCE induces filopodia in the absence of an apparent Ca^2+^-binding structural element. **a** During ER depletion, TRPC1 (red) retreats to internal membrane compartments (middle frame). Although [Ca^2+^]_i_ is elevated, Ca^2+^ flux through Orai is impossible because there is no extracellular Ca^2+^. Upon Ca^2+^ addition, the TRPC-bearing vesicles undergo exocytosis. **b** Proposed mechanism of vesicle exocytosis. Vesicles are attached to microtubules and mobilized because of the Ca^2+^ influx through Orai channels. The lack of Orai alignment in linear arrays (data not shown) suggests that Orai is not mobilized to the plasma membrane in the same way as TRPC1. **c** Putative mammalian signalplex underlies TRPC1 localization and dissemination. On the left side, TRPC1 (red), scaffold protein (brown), and ERM (gray and green) form an aggregate in the plasma membrane on a substrate of actin (blue). Upon Ca^2+^ influx, TRPC channels dissociate from the scaffold (right side)

As highly selective channels, Orai would only allow influx after Ca^2+^ addition. We propose that the choreographed movements of TRPC vesicles after Ca^2+^ readdition are explained by the function of Orai channels, which is supported by previous evidence that Orai works in cooperation with TRPC-bearing vesicles [17]. Interestingly, this mechanism may be conserved in the evolutionary sense, as sustained Ca^2+^ influx had caused the translocation of a canonical Drosophila TRP channel to the plasma membrane [82]. Ca^2+^ influx may affect secretion directly or, for those vesicles arrayed on microtubules, may promote their microtubule-dependent trafficking to the cell surface. Our model (Fig. 10) has the attractive feature that the concentration of vesicles from the RRP in the vicinity of Orai ensures that they can respond to a rapid, local elevation of Ca^2+^.

#### Role of PLC in TRPC activation

Some concentration of PI(4,5)P2 was essential to activate TRPC channels [71], but its relationship to channel function is complex (see for review [80]). It has been proposed that the local generation of diacylglycerol activates TRPC1/4/5 [98]. Our evidence is more consistent with the known mechanism of gating by STIM1 [121], as DOG had little effect, but TRPC1-STIM1 correlation coefficients were elevated after Ca^2+^ readdition. Gating by a scaffold molecule, such as NHERF, is also possible [30]. Nevertheless, PI(4,5)P2 turnover was permissive both for TRPC5 activation and for its inhibition by PKC downstream of G protein (Gq/11) stimulation [73]. In membranes of the *Drosophila* photoreceptor, the activation of PLC led to TRPL activation simply by converting PI(4,5)P2 to diacylglycerol with release of a proton, which neutralizes the acidic character of the membrane and changes its curvature [40]. This gating mechanism has the virtue of simplicity but cannot account for all the findings in the current experiments. With the elevation of [Ca^2+^]_i_, Ca^2+^ binding to PLC may cause a conformational change and expose sites for docking molecules activated by signaling from G protein-coupled receptors and tyrosine kinase receptors (see for review [9]). As mentioned above (see “TRPC localization and activity follow a bell-shaped pattern” section), the media used were devoid of ligands that could initiate upstream signaling. PLCβ was not thought to be activated by Ca^2+^ directly (see below) but may be activated by binding a Rho family GTPase. Secondly, PI(4,5)P2 serves to nucleate actin filaments by activating profilactin [51] and by seeding ezrin binding sites, which recruit I-BAR proteins such as IRSp53 [106], (see for review [25]). In most cells, I-BAR proteins are fundamental constituents of filopodia [2, 25, 37]. Thus, PI(4,5)P2 depletion should have counteracted filopodia formation, i.e. replicated the effects of SKF96365 and pico145 (Fig. 7a, b), but the evidence indicated that Ca^2+^ influx enabled filopodia extension.

As suggested above (see “Anchorage in the signalplex and cytoskeleton” section), a useful model for TRPC activation may be the *Drosophila* signalplex, which organizes a number of signaling proteins on a scaffold protein containing multiple PDZ motifs, called INAD (inactivation-no-afterpotential-D). INAD is attached to the plasma membrane by TRP/TRPL, the only transmembrane proteins in the complex. *Drosophila* orthologues of PKC and PLCβ are recruited to additional PDZ motifs of INAD. The structure of the putative mammalian signalplex is similar, although assembled on NHERF which has fewer PDZ motifs. During ER depletion, TRPC1 clusters resembled a sort of signalplex, suggesting that channels in this form were inactive. Nevertheless, the colocalization shows that they were in close proximity to PLC. Because clusters were dispersed after Ca^2+^ readdition, TRPC was simultaneously activated and detached from the scaffold. This is consistent with previous reports that PI(4,5)P2 enhanced the formation of the mammalian TRPC1/4 and TRPC1/5 complexes (see for review [90]), but inhibited their activation [74]. PI(4,5)P2 cleavage caused a conformational change at the C-terminus of TRPC4/5, resulting in its dissociation from NHERF and simultaneously causing channel opening [90]. Despite channels being activated downstream of PI(4,5)P2 hydrolysis, the biological effect (light-sensing) of activation in the *Drosophila* photoreceptor was short-lived in TRPL mutants. If PI(4,5)P2 breakdown was maximized, it was rapidly depleted, which terminated TRP channel activation and Ca^2+^ influx. This was attributed to the absence of Ca^2+^-dependent inhibition of PLC by Ca^2+^ influx through TRP channels [33], (see for review [19]).

The interpretation of previous work was complicated by the fact that the cleavage product of PLC, IP_3_, is itself an activator of TRPC channels through SOCE. Because the current experiments show that a long time elapsed between SOCE induction and the stimulus for filopodia extension, these signaling pathways are not considered influential in extension. The turnover of PI(4,5)P2 appears to be regulated by opposing effects of Ca^2+^ on PLCβ. Whereas it is not clear that the PLCβ isoforms are Ca^2^-activated, Ca^2+^ stimulates the in vitro catalytic activity of PLCδ and η over 2–3 orders of magnitude [72]. The EF-hands of PLCβ3 do not appear to bind Ca^2+^ but rather cause a conformational change that may activate the GTPase-activating activity of PLC, thereby releasing the bound GTPase (see for review [31]). A similar braking action, mediated by Ca^2+^ in the *Drosophila* photoreceptor system, allows the substrate levels to be replenished and extends the duration of the biological effect [33]. As the local PI(4,5)P2 molecules are degraded but the PI(4,5)P2 concentration is maintained, this process resembles treadmilling. Unlike the well-known treadmills of the cytoskeleton, it pertains to a membrane-bound structure. If replenishment comes from a membrane-bound vesicle, and especially if it has a directional component, it might more properly be called a conveyor belt.

#### TRPC and actin cytoskeleton

As noted above (see “Ca^2+^ readdition stimulates TRPC secretion” section), dissemination of TRPC1 throughout the cell surface was closely related to filopodia extension. Channel gating, by whatever means (see “Role of PLC in TRPC activation” section), does not explain their dissemination. It should be noted that, in the putative mammalian signalplex, NHERF is recruited to TRPC4/5 and in turn recruits ERM (see “Anchorage in the signalplex and cytoskeleton” section). Moreover, the PLCβ isoenzymes bind to NHERF and turn over PI(4,5)P2. As ERM proteins are ubiquitous organizers of the subcortical actin filaments, the signalplex may be supported by an actin network. ERM proteins are also bound to the plasma membrane via PI(4,5)P2 (see for review [41]) and are released by stimuli leading to PLC-β activation and PI(4,5)P2 breakdown [18]. If ERM acts as a latchhook on the structure, its dissociaton could release it from anchorage in the putative signalplex. While previous reports have shown TRPC5/6 mobilization immediately after receptor ligation [8, 70], we are unaware of any showing SOCE-induced mobilization of channels from clusters. However, it should be noted that PLC, scaffold protein, and ERM do not assemble as unitary elements. As TRPC channels assemble as tetramers and PLCβ as a dimer, a single tetramer could bind as many as four NHERF2 scaffold proteins, and a dimer as many as two. There are differing reports of the PDZ motifs to which isoenzymes of PLCβ are bound, so it is uncertain whether TRPC and PLC can be assembled on a single molecule. If the whole structure, illustrated in Fig. 10c, employed ERM to attach to multiple actin filaments, it might be dissolved concurrently with TRPC channel activation. This model has the advantage of explaining how diacylglycerol and NHERF both appear to gate the TRPC channels (see “Role of PLC in TRPC activation” section). Future studies of the signalplex constituents, i.e. TRPC, PLC, the scaffold, ERM, and actin, using high-resolution imaging, are needed to dissect the relationship between TRPC channel dissemination and the structure of this putative mammalian signalplex.

### Polarity and Ca^2+^ influx

As mentioned above, vesicles from the RRP fuse with the plasma membrane depositing additional TRPC1 channels on the cell surface (see “Exocytosis and stimulus-coupled secretion during extracellular Ca^2+^ replenishment” section). This was consistent with previous evidence implicating Rab4-dependent recycling in the exchange of TRPC vesicles between intracellular compartments and the plasma membrane [17, 21]. Here, it is noteworthy that SOCE-related STIM migration to the cell surface, Orai activation, and exocytosis of TRPC-bearing vesicles remained intact despite the absence of signaling from hormones or growth factors. SOCE-related Ca^2+^ influx likely occurred over the entire cell surface under the current experimental conditions. However, under conditions of receptor-ligand interaction, signaling would have directional significance and thus, both STIM migration and TRPC1 exocytosis may have a directional bias.

### TRPC activity and mechanosensory function

TRP channels regulate many different kinds of sensory transduction, including pheromone signaling, taste transduction, and temperature and pain sensation. TRPC1 is implicated directly in transduction, as it can be activated by mechanical stretch in reconstituted liposomes [63]. Our evidence suggested that translocation of STIM and TRPC1 to the plasma membrane were important determinants of filopodia dynamics. Both underwent redistribution during each phase of two-stage SOCE induction, in contrast to Orai and several proteins unrelated to SOCE. The TRP channels associated with filopodia extension were activated and unanchored, suggesting that the role of TRPC channel activation was to ensure Ca^2+^ influx. What is the role of Ca^2+^ influx? The above suggests that the downstream effector of the activated channel is PLC, and Ca^2+^ influx through TRPC1/4/5 acts to brake its activity. This allows PI(4,5,)P2 renewal by “PIP2 treadmilling” and assures a steady supply of the molecules required for filopodia extension. Some proteins that participate in filopodia assembly, for example the WAVE complex, Enabled, and IRSp53, were bound to PI(4,5)P2 (see for review [5, 37, 55, 111]). Although TRPC and PLC were colocalized in spots on the filopodia, and the recruitment of such proteins may depend on these sites, they were not confined to the filopodia.

The life cycle of the filopodium comprises four phases, namely initiation, extension, persistence, and retraction. The evidence suggested that extension was the phase regulated by Ca^2+^ influx. Initiation typically took place at sites of actin assembly and required activation of myosin X by PI(3,4,5)P3 [34]. If wortmannin had blocked PI(3,4,5)P3 synthesis, initiation would have been inhibited. This was not found, suggesting that, throughout ER depletion, the filopodia were primed to undergo extension. What is the role of TRPC-bearing vesicle secretion? One intriguing speculation is that, by adding to the membrane, exocytosis reduces the force exerted by the subcortical actin network. Centripetal forces on the plasma membrane, maintained by the pushing force of the actin network and in-plane tension of the lipid bilayer, limit the cell’s ability to form protrusions. For filopodia extension, this must be overcome by an opposing force of ~ 60 pN, which is generated by polymerization of the actin filaments that make up their core (see for reviews [5, 37, 55, 111]).

Both ERM dissociation from the plasma membrane and exocytosis from the RRP might be able to disrupt the subcortical network, so that either may create sites for rearranging the existing filaments or assembling new filaments. It has been proposed that there is a protein that “seeds” patches of membrane for filopodia extension (see for review [25]). Although such a protein has not been identified, it may be associated with the RRP vesicles. The additional constituents that we identified as being partially colocalized with TRPC1, namely AQP4 and Vamp2, were not obvious mediators of a “seed” function. However, it is possible that a PI(4,5)P2 precursor, or PI(4,5)P2 itself, is delivered to the cell surface in these vesicles. The need to maintain a supply of PLC substrate, to keep pace with its breakdown, would be consistent with the need for recycling from the RRP. As the biochemical precursor of PI(4,5)P2 was processed by PI4P 5-kinase [33], this would be another candidate. In either case, the “seed” role would be satisfied by any molecule(s) that ensured “PIP2 treadmilling”.

## Supplementary Information

**Additional file 1:** Supplementary Figures and Tables.

## Data Availability

The datasets used and/or analyzed during the current study are available from the corresponding author on reasonable request.

## Abbreviations

AQP4: Aquaporin isoform
CALP2: Calcium-like peptide 2
CaMKII: Ca^2+^/calmodulin-dependent kinase II
Ca^2+^-free HBSS: Ca^2+^-Mg^2+^-free Hanks’ balanced salt solution
CPA: Cyclopiazonic acid
CRAC: Ca^2+^ release-activated Ca^2+^ channel
CaV1.2: Channel subunit of VACC
EF-hand: A calcium-binding motif
ER: Endoplasmic reticulum
ERM: Ezrin: radixin: and moesin
FITC: Fluorescein isothiocyanate
HBSS: Hanks balanced salt solution
IP_3_: Inositol 1,4,5 trisphosphate
IP_3_R: IP_3_ receptor
MLCK: Myosin light chain kinase
NHERF: Na^+^/H^+^ exchanger regulatory factor
PI: Phosphoinositide
PI(4,5)P2 (PIP2): Phosphatidylinositol 4,5 bisphosphate
PI3-K: Phosphoinositide 3-kinase
PKA: Cyclic AMP-dependent protein kinase
PKC: Protein kinase C
PLC: Phospholipase C
PTEN: Phosphatase and TENsin homolog deleted on chromosome 10
RRP: Rapidly releasable pool
SOCE: Store-operated calcium entry
STIM: Stromal-interacting molecule
TRPC1: Canonical transient receptor potential channel 1
Vamp2: Vesicle-associated membrane protein 2
VACC: Voltage-activated calcium channel
WAVE: WASP (Wiskott-Aldrich syndrome protein) family verprolin-homologous protein
