# Supplementary material for: How filopodia respond to calcium in the absence of a calcium-binding structural protein: non-channel functions of TRP
Source: Cell Commun Signal. 2022 Aug 26;20:130. doi: 10.1186/s12964-022-00927-y (PMC9414478; doi:10.1186/s12964-022-00927-y)
Supplement: Supplementary file 2 — Additional file 1: Supplementary Figures and Tables. [file 12964_2022_927_MOESM2_ESM.docx]

# Supplementary Figures and Tables

## Supplementary Figures


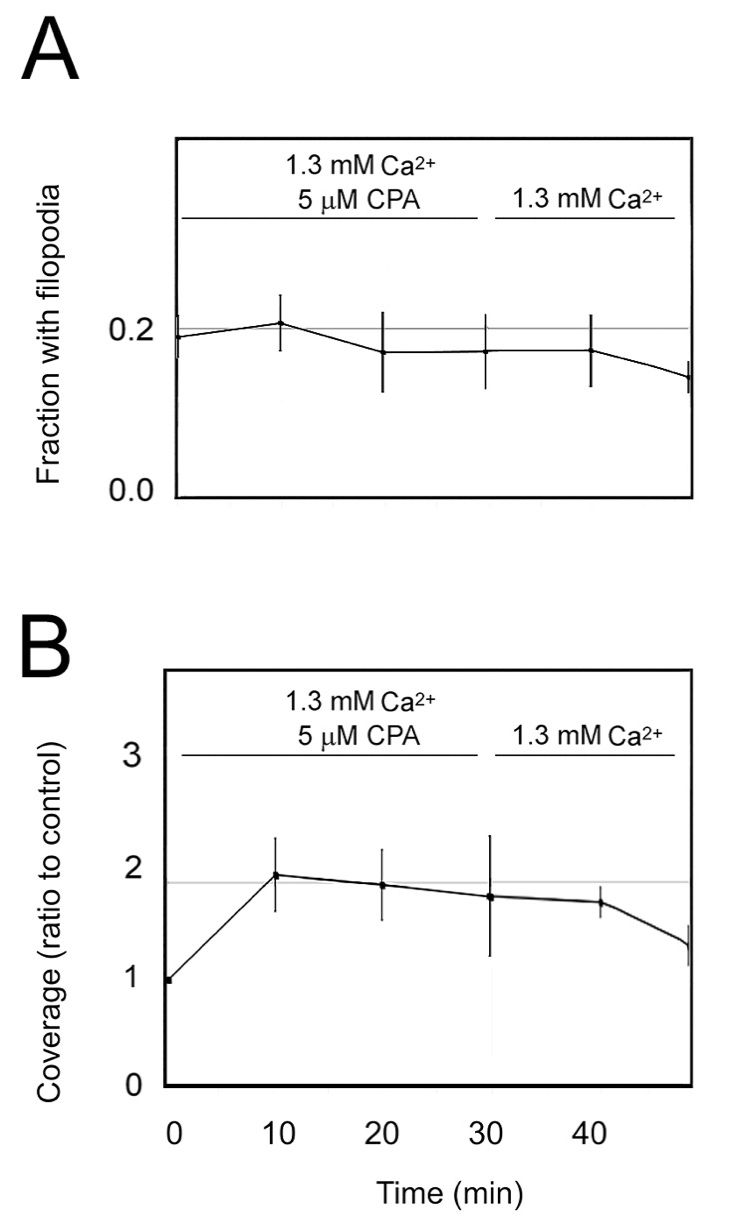


**Supplementary Figure 1.** Filopodia prevalence after CPA treatment in Ca^2+^-replete HBSS. Cells were exposed to 5 μM CPA in HBSS containing Ca^2+^. (A) The number of cells showing filopodia changed little during the procedure. Significance of differences by ANOVA, P=0.140. (B) The percentage of the cell edge occupied by filopodia increased immediately after CPA treatment but underwent little change after CPA washout. Significance of experiment by ANOVA, P=0.300.


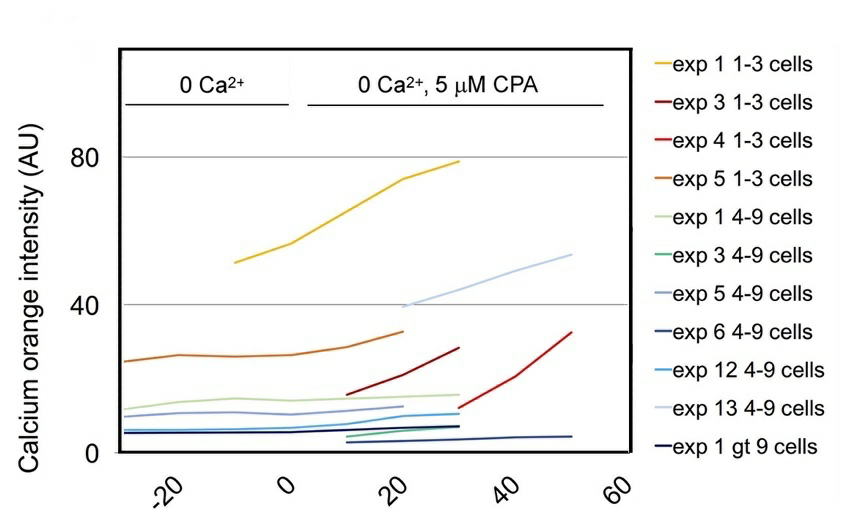


**Supplementary Figure 2.** Fluorescence of cells preloaded with calcium indicator and then exposed to Ca^2+^-free HBSS and subsequently CPA. CPA was added at time=0 and the sample was loaded and imaged as rapidly as possible to record the fluorescence intensity. Intensity is shown as the moving average of two time points to eliminate short-term fluctuations. As only single cells were evaluated for filopodia, we compared 1-3 cells (yellow and red) and colonies of cells (blue and green) to determine whether the changes in Ca^2+^ were uniform. These areas differed in the amounts of Ca^2+^ they accumulated. Significance of experiment by ANOVA repeated measures, P<0.0001.

**
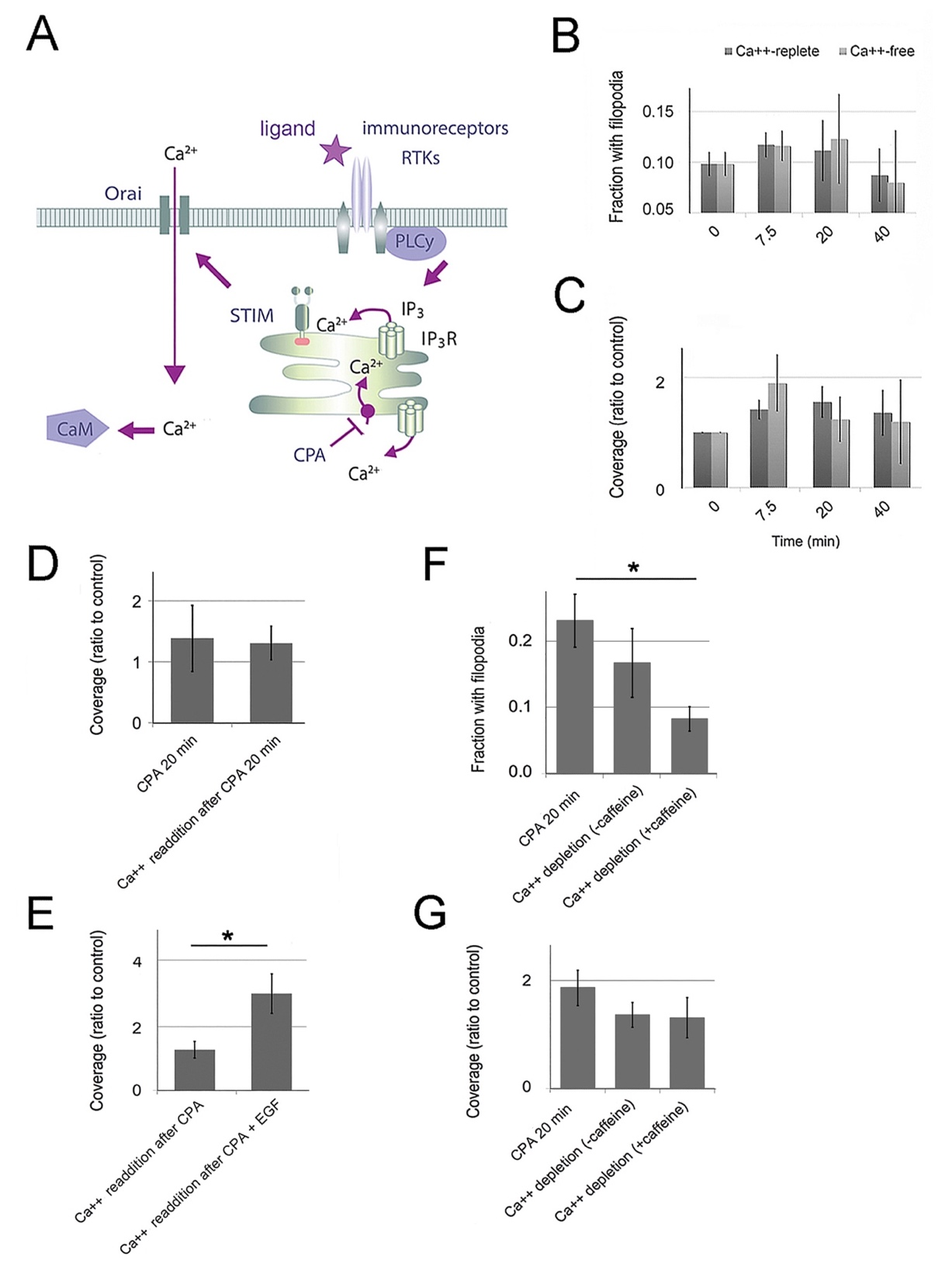
**

**Supplementary Figure 3.** SOCE initiation leads to changes in filopodia prevalence. (A) SOCE induction downstream of ligand-receptor interactions (star). Inositol 1,4,5-trisphosphate (IP_3)_ production is initiated by activation of receptor tyrosine kinases (RTKs) upstream of phospholipase Cγ (PLCγ). Release of Ca^2+^ through the IP_3_ receptor (IP_3_R), which is a Ca^2+^ channel, depletes the ER of Ca^2+^ and activates STIM. CPA prevents Ca^2+^ uptake by the SERCA pump (●), preventing its reentry into the ER, so it accumulates in the cytoplasm. (B-C) Prevalence after replacement of the media with Ca^2+^-replete or Ca^2+^-free HBSS. One-way ANOVA on the experiments had P values from 0.469 to 0.759. (D) Cells were exposed for 20 minutes to 5 μM CPA in Ca^2+^-free HBSS and then fixed immediately (CPA 20 min) or washed with Ca^2+^-free HBSS and incubated in Ca^2+^-replete HBSS (Ca^2+^ readdition after CPA 20 min). (E-G) Prevalence analyzed after 5 μM CPA treatment for 20 minutes in Ca^2+^-free HBSS in the presence or absence of the designated agent, followed by CPA washout and readdition of Ca^2+^. (E) The coverage of the cell edge after Ca^2+^ readdition is enhanced if EGF was present during ER depletion (cf. Figure 1A-B). Results shown are from 4 experiments. *Treatments differed at P=0.033. (F) The number of cells showing filopodia after Ca^2+^ readdition is decreased after ER depletion with 150 mM caffeine included during ER depletion. ANOVA on experiment, P=0.032, *Treatments differed at P=0.026. (G) The coverage of cells with filopodia did not depend on whether 150 mM caffeine was present or absent during depletion. ANOVA on the experiment, P=0.270. Bars represent ± standard error of the mean (S.E.M.).


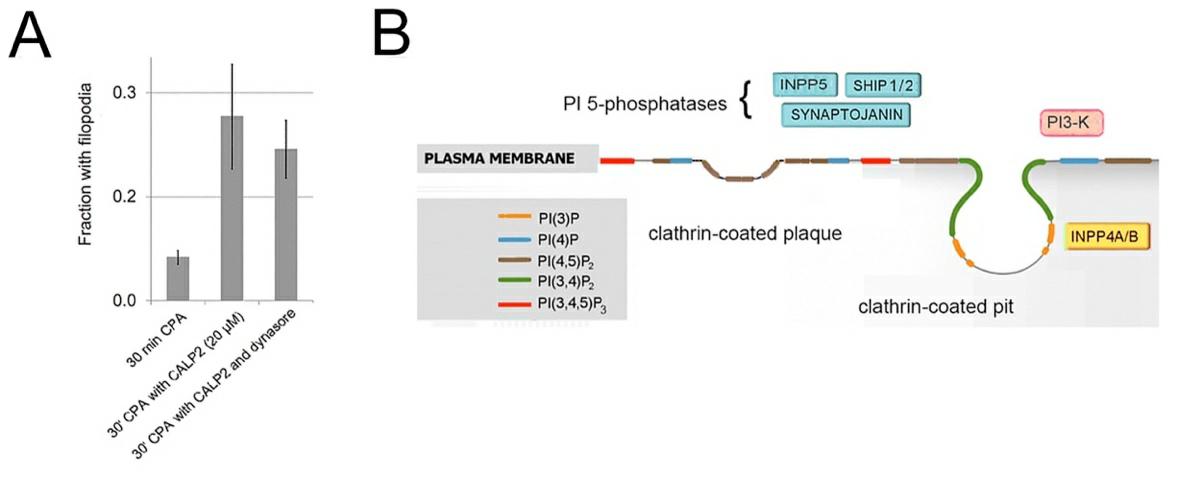


**Supplementary Figure 4.** Contribution of endocytosis to filopodia rescue. (A) Fraction of cells showing filopodia after treatment with CPA alone or with CPA and 20 μM CALP2 in the presence or absence of 45 μM dynasore. ANOVA on the experiment, P=0.00027. (B) Chemotactic cues stimulate the accumulation of phosphatidylinositol 3,4,5-trisphosphate (PI(3,4,5)P3). The substrate of PI3-K, phosphatidylinositol 4,5-bisphosphate (PI(4,5)P2), is found in the plasma membrane especially at clathrin-coated plaques. The product, (PI(3,4,5)P3), is converted to PI(3,4)P2 by PI 5-phosphatases. PI(3,4)P2 participates in endocytic vesicle maturation. PI(3,4)P2 can also be formed by phosphorylation of PI(4)P in an alternate pathway (Schmid, 2014;Stahelin et al., 2014;Bilanges et al., 2019;Dickson and Hille, 2019;Sugiyama et al., 2019;Gozzelino et al., 2020). INPP5=PI(4,5)P2 5-phosphatase; SHIP1/2=SH-2 containing inositol 5' polyphosphatase; Synaptojanin=5-phosphatase employing PI(3,4,5)P3 and PI(4,5)P2 as substrates

**Targets of Ca^2+^/calmodulin**

Because [Ca^2+^]_i_ increased continuously, as shown in Figure 1C, its effect on filopodia dynamics may depend on the channel proteins’ binding to calmodulin. It has been reported that calmodulin antagonists potentiate SOCE (Galán et al., 2011), and indeed, calmodulin is thought to be constitutively bound to many TRP channels. The Ca^2+^/calmodulin could then downregulate the channel’s activity through Ca^2+^-dependent inactivation (Li et al., 2017), (see for review (Saimi and Kung, 2002;Singh et al., 2002;Bezzerides et al., 2004;Gees et al., 2010;Wang et al., 2020). However, *in vitro* studies suggested that Ca^2+^ levels exceeding 10^-5^ M were required for Ca^2+^/calmodulin binding to TRPC4 channels (Trost et al., 2001). Whereas inactivation by CDI would have prevented influx under normal conditions, extracellular Ca^2+^ was not present during ER depletion. Thus, inhibition of Ca^2+^ influx was not a possible mechanism. TRPC5 opening was unusual in being potentiated by elevated intracellular Ca^2+^ levels, rather than inhibited (Blair et al., 2009). It was anticipated that TRPC5 channel opening would be replicated by treating the cells with (-)-englerin, but this had no effect on filopodia prevalence.

Another possible mechanism was Ca^2+^/calmodulin competition at a PI(3,4,5)P3 binding site on TRPC6. Ca^2+^/calmodulin binds to a C-terminal site, inhibiting the channel, and competition for the site by PI(3,4,5)P3 increased the current through the channel (Kwon et al., 2007). Under this mechanism, wortmannin would inhibit PI(3,4,5)P3 production and TRPC6 activity, but this would give the opposite results of the wortmannin effect observed in the current experiments (Figure 4E-F).

Wortmannin also inhibited MLCK, and so we entertained the possibility that this mechanism was responsible for its ability to increase filopodia. Again, the blockade of protrusion extensions was expected, but wortmannin had the opposite effect. As MLCK inhibitors, altenusin, MLCK peptide, and ML-7, had little effect over CPA alone (data not shown), MLCK inhibition was ineffective over timespans as short as those used in the current experiments. Wortmannin also inhibited TRPC5-mediated [Ca^2+^]_i_ rises in a concentration-dependent manner (Shimizu et al., 2006).

Two additional mechanisms of CALP2-mediated enhancement were considered. The binding site for Ca^2+^/calmodulin on Orai overlaps its binding site for STIM (Bhardwaj et al., 2020). If there had been an increase in Ca^2+^/calmodulin, as suggested by the results of Figure 1C, STIM1 binding to Orai at the plasma membrane could be blocked inhibiting formation of a STIM1-Orai complex. On the contrary, STIM1 became colocalized with Orai on the plasma membrane (see **3.1.6 Loci containing STIM1, Orai, TRPC1, AQP4, and Vamp2 during ER depletion**), suggesting that any presumed elevation in Ca^2+^/calmodulin did not block STIM1-Orai complex. Lastly, all TRPC isoforms shared a C-terminal calmodulin- and IP_3_R-binding site, where Ca^2+^/calmodulin competes with IP_3_R (see for review (Zhu, 2005)). There was a possibility that Ca^2+^/calmodulin dissociated TRPC channels from membrane-bound IP_3_R sites. However, TRPC1-STIM1 colocalization was unchanged during ER depletion when [Ca^2+^]_i_ was maximal (see **3.1.6 Loci containing STIM1, Orai, TRPC1, AQP4, and Vamp2 during ER depletion**). It was only increased after Ca^2+^ readdition, when [Ca^2+^]_i_ was declining (cf. Figure 1D and Table 2, Supplementary Figure 4C).

**
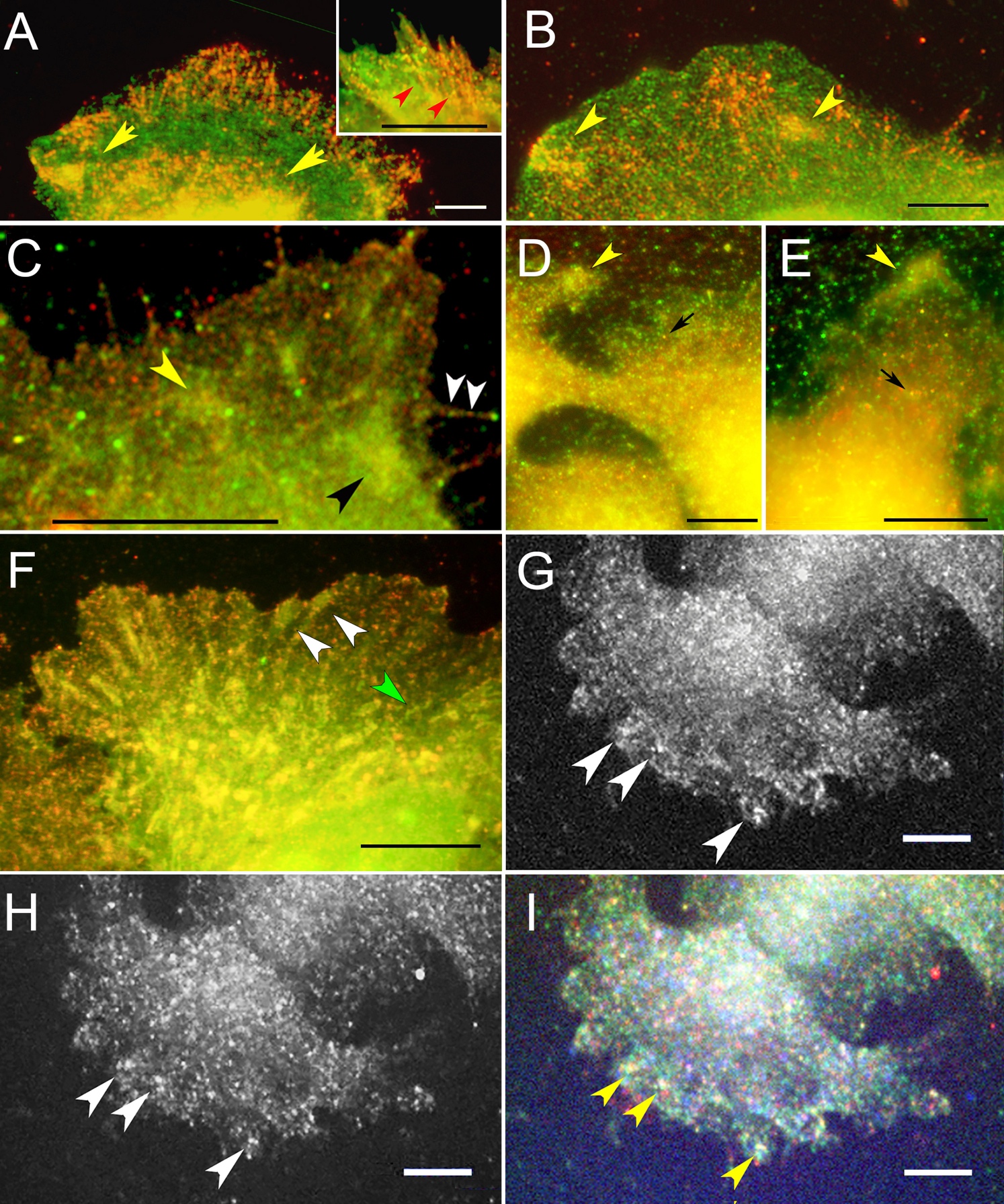
Supplementary Figure 5.** SOCE mediators during ER depletion and after Ca^2+^ readdition. (A, B) STIM1 (red) with Orai (green) in amorphous patches (yellow arrowheads). (A) STIM1-Orai1 coincidence (arrowheads) in a cell after ER depletion. Inset: Orai3-STIM1 colocalization showing STIM punctae in linear arrays (red arrowheads), (B) STIM1-Orai1 coincidence (arrowheads) in a cell after Ca^2+^ readdition, (C) TRPC1 (green) and STIM1 (red) alternating in location on filopodia (white arrowheads) and diffusely distributed on the cell surface (yellow and black arrowheads) after Ca^2+^ readdition, (D-F) TRPC1 (green) and Vamp2 (red) colocalization. TRPC1 and Vamp2 are not commonly together within the same vesicles (black arrows) but coincide at elevated portions of the cell (yellow arrowheads). (D) Vamp2-containing particles at the edge of a cell after Ca^2+^ readdition. (E) TRPC1-containing particles at the edge of a cell after Ca^2+^ readdition. (F) Colocalization of integrin β1 (green) and TRPC1 (red). Integrin is found in cytoskeletal structures (white arrowheads) and tubular profiles in the cytoplasm (green arrowhead), while TRPC1 is localized to vesicles. (G-I) Confocal planes from cells stained with anti-STIM1 (blue), Orai (green), and TRPC1 (red). (G, H) Orai and TRPC1, respectively, in a ventral plane near the lamella (arrowheads), (I) STIM1-Orai (cyan) and TRPC1-Orai (yellow) coincide during ER depletion. Orai1 and Orai3 showed no difference in the pattern of colocalization with STIM1. Bars =10 µm


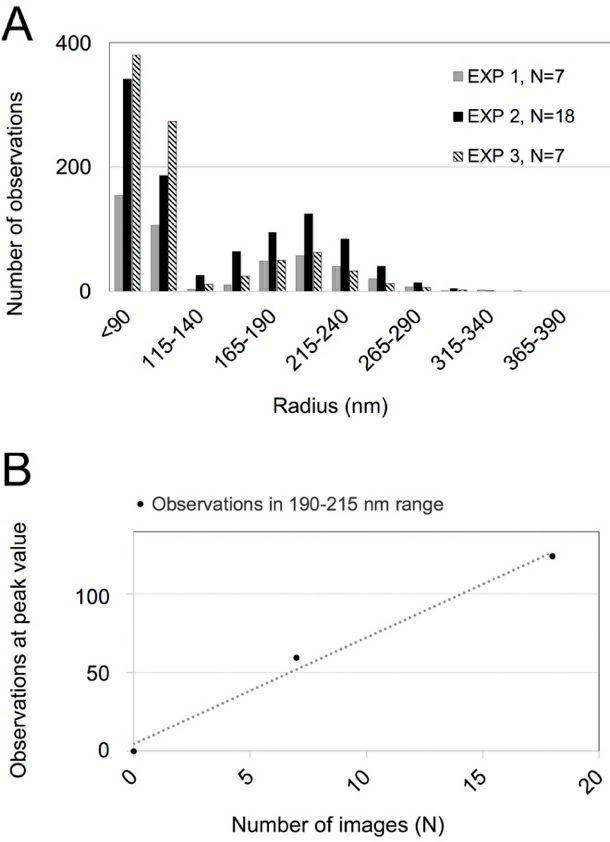


**Supplementary Figure 6.** Number of particles recovered from segmented images and their relationship to the number of images sampled. (A) Distribution of particles retrieved from segmented images of TPRC1. (B) Linear relationship between the number of images analyzed and the number of particles recovered.


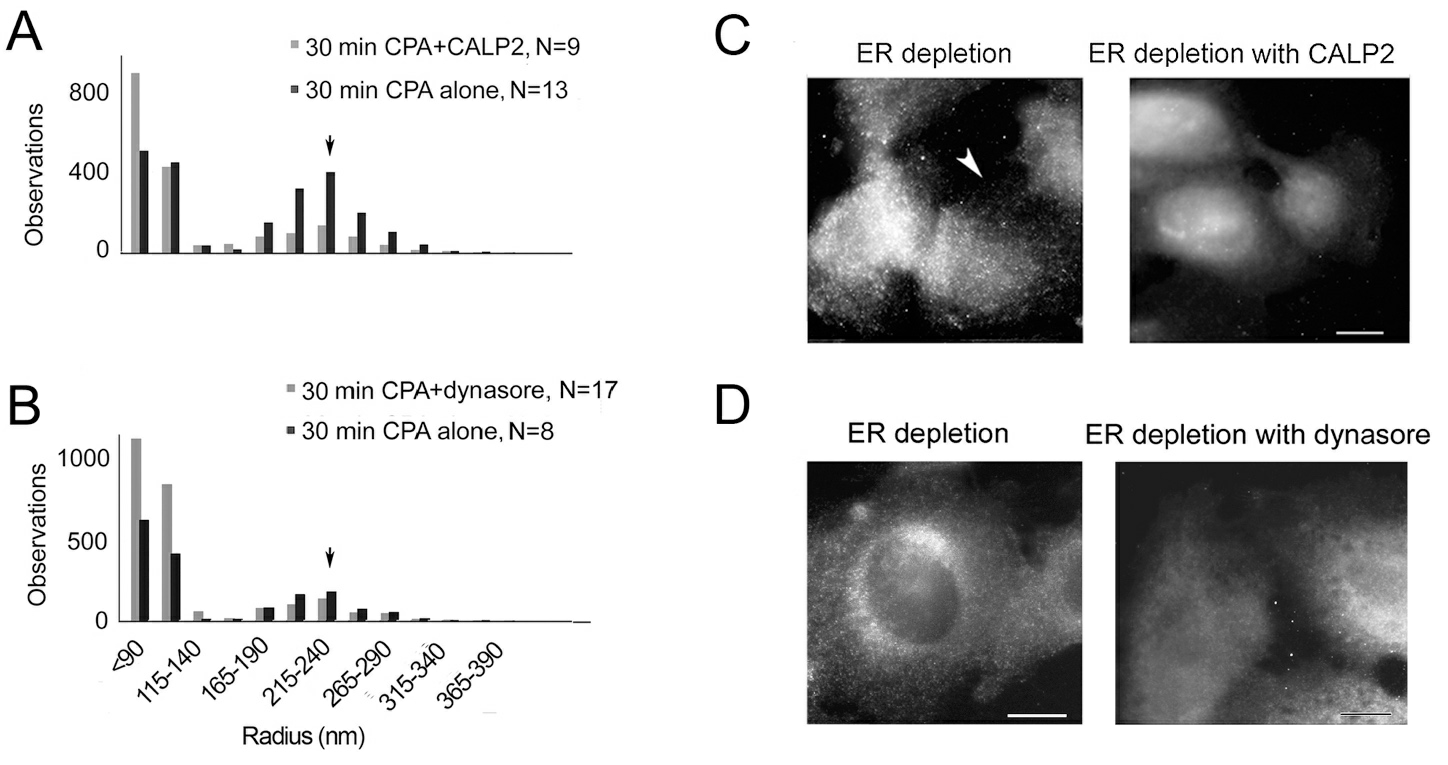


**Supplementary Figure 7.** Surface TRPC1 localizations in the presence or absence of agents stimulating filopodia extension during ER depletion. (A-B) TRPC1-bearing loci were localized at the surface of cells that were not permeabilized, and those with a circular form were subjected to analysis (see **2.10 Analysis of particle sizes after staining by indirect immunocytochemistry**). Comparing their distributions during ER depletion showed that particles with radii ~215 nm were well-represented after CPA alone (cf. Figure 5C), but radii <140 nm predominated in the treated cells. (C-D) Cells were fixed but not permeabilized, and then localization procedures were performed to detect TRPC1 with antibody against the extracellular domain (see **2.6 Immunofluorescence localization, image acquisition, and image processing**). (C) Cells depleted in the presence or absence of 20 µM CALP2. (D) Cells depleted in the presence or absence of 48 µM dynasore. Bars =10 µm

## Supplementary Tables

Table S1. Protein pairs with unchanged correlation coefficients in three SOCE phases*

| Phase | STIM1-Orai | STIM1-AQP4 | TRPC1-caveolin | TRPC1-Orai | TRPC-CaV1.2 |
| --- | --- | --- | --- | --- | --- |
| 1 | 0.51 (0.04^†^, N=10^‡^) | 0.70 (0.06, N=10) | 0.49 (0.08, N=7) | 0.52 (0.04, N=7) | 0.55 (0.03, N=10) |
| 2 | 0.55 (0.07, N=6) | 0.82 (0.08, N=7) | 0.47 (0.06, N=6) | 0.60 (0.04, N=8) | 0.64 (0.03, N=9) |
| 3 | 0.57 (0.04, N=18) | 0.75 (0.07, N=5) | 0.59 (0.08, N=5) | 0.52 (0.04, N=11) | 0.63 (0.04, N=7) |

*Phase 1 untreated; phase 2 ER depletion; phase 3 Ca^2+^ readdition

^†^S.E.M.

^‡^number of images

## References

Bezzerides, V.J., Ramsey, I.S., Kotecha, S., Greka, A., and Clapham, D.E. (2004). Rapid vesicular translocation and insertion of TRP channels. *Nat Cell Biol* 6**,** 709-720.

Bhardwaj, R., Augustynek, B.S., Ercan-Herbst, E., Kandasamy, P., Seedorf, M., Peinelt, C., and Hediger, M.A. (2020). Ca^2+^/calmodulin binding to STIM1 hydrophobic residues facilitates slow Ca^2+^-dependent inactivation of the Orai1 channel. *Cell Physiol Biochem* 54**,** 252-270.

Bilanges, B., Posor, Y., and Vanhaesebroeck, B. (2019). PI3K isoforms in cell signalling and vesicle trafficking. *Nat Rev Mol Cell Biol* 20**,** 515-534.

Blair, N.T., Kaczmarek, J.S., and Clapham, D.E. (2009). Intracellular calcium strongly potentiates agonist-activated TRPC5 channels. *J Gen Physiol* 133**,** 525-546.

Dickson, E.J., and Hille, B. (2019). Understanding phosphoinositides: rare, dynamic, and essential membrane phospholipids. *Biochem J* 476**,** 1-23.

Galán, C., Dionisio, N., Smani, T., Salido, G.M., and Rosado, J.A. (2011). The cytoskeleton plays a modulatory role in the association between STIM1 and the Ca channel subunits Orai1 and TRPC1. The cytoskeleton plays a modulatory role in the association between STIM1 and the Ca channel subunits Orai1 and TRPC1. *Biochem Pharmacol* 82**,** 400-410.

Gees, M., Colsoul, B., and Nilius, B. (2010). The role of transient receptor potential cation channels in Ca^2+^ signaling. *Cold Spring Harb Perspect Biol* 2**,** a003962.

Gozzelino, L., De Santis, M.C., Gulluni, F., Hirsch, E., and Martini, M. (2020). PI(3,4)P2 signaling in cancer and metabolism. *Front Oncol* 10**,** No 360; [https://doi.310.3389/fonc.2020.00360](about:blank).

Hasan, R., Leeson-Payne, A.T., Jaggar, J.H., and Zhang, X. (2017). Calmodulin is responsible for Ca^2+^-dependent regulation of TRPA1 channels. *Sci Rep* 7**,** 45098.

Kwon, Y., Hofmann, T., and Montell, C. (2007). Integration of phosphoinositide and calmodulin mediated regulation of TRPC6. *Mol Cell* 25**,** 491-503.

Lee, M.Y., Song, H., Nakai, J., Ohkura, M., Kotlikoff, M.I., Kinsey, S.P., Golovina, V.A., and Blaustein, M.P. (2006). Local subplasma membrane Ca^2+^ signals detected by a tethered Ca^2+^ sensor. *PNAS U. S. A.* 103**,** 13232-13237.

Lewis, R.S. (2011). Store-operated calcium channels: new perspectives on mechanism and function. *Cold Spring Harb Perspect Biol,3(12), a003970.* doi: 10.1101/cshperspect.a003970

Li, X., Wu, G., Yang, Y., Fu, S., Liu, X., Kang, H., Yang, X., Su, X.-C., and Shen, Y. (2017). Calmodulin dissociates the STIM1-Orai1 complex and STIM1 oligomers. *Nat Comm* 8**,** no. 1042.

Pulina, M.V., Zulian, A., Berra-Romani, R., Beskina, O., Mazzocco-Spezzia, A., Baryshnikov, S.G., Papparella, I., Hamlyn, J.M., Blaustein, M.P., and Golovina, V.A. (2010). Upregulation of Na+ and Ca^2+^ transporters in arterial smooth muscle from ouabain-induced hypertensive rats. *Am J Physiol: Heart Circ Physiol* 298**,** H263-H272.

Saimi, Y., and Kung, C. (2002). Calmodulin as an ion channel subunit. *Ann Rev Physiol* 64**,** 289-311.

Schmid, S.L. (2014). *Endocytosis.* Cold Spring Harbor, NY.

Shimizu, S., Yoshida, T., Wakamori, M., Ishii, M., Okada, T., Takahashi, M., Seto, M., Sakurada, K., Kiuchi, Y., and Mori, Y. (2006). Ca^2+^–calmodulin-dependent myosin light chain kinase is essential for activation of TRPC5 channels expressed in HEK293 cells. *Journal Physiology* 570**,** 219-235.

Singh, B.B., Liu, X., Tang, J., Zhu, M.X., and Ambudkar, I.S. (2002). Calmodulin regulates Ca^2+^-dependent feedback inhibition of store-operated Ca^2+^ influx by interaction with a site in the C terminus of TrpC1. *Mol Cell* 9**,** 739-750.

Stahelin, R.V., Scott, J.L., and Frick, C.T. (2014). Cellular and molecular interactions of phosphoinositides and peripheral proteins. *Chem Phys Lipids* 182**,** 3-18.

Sugiyama, M.G., Fairn, G.D., and Antonescu, C.N. (2019). Akt-ing up just about everywhere: Compartment-specific Akt activation and function in receptor tyrosine kinase signaling. *Front Cell Devel Biol* 7**,** 70.

Tang, J., Lin, Y., Zhang, Z., Tikunova, S., Birnbaumer, L., and Zhu, M.X. (2001). Identification of common binding sites for calmodulin and inositol 1,4,5-trisphosphate receptors on the carboxyl termini of Trp channels. *J Biol Chem* 276**,** 21303-21310.

Tóth, A., Fodor, J., Vincze, J., Oláh, T., Juhász, T., Zákány, R., Csernoch, L., and Zádor, E. (2015). The effect of SERCA1b silencing on the differentiation and calcium homeostasis of C2C12 skeletal muscle cells. *PLoS ONE* 10**,** e0123583.

Trost, C., Bergs, C., Himmerkus, N., and Flockerzi, V. (2001). The transient receptor potential, TRP4, cation channel is a novel member of the family of calmodulin binding proteins. *Biochem J* 355**,** 663-670.

Vinayagam, D., Quentin, D., Yu-Strzelczyk, J., Sitsel, O., Merino, F., Stabrin, M., Hofnagel, O., Yu, M., Ledeboer, M.W., Nagel, G., Malojcic, G., and Raunser, S. (2020). Structural basis of TRPC4 regulation by calmodulin and pharmacological agents. *Elife* 9**,** e60603.

Wang, H., Cheng, X., Tian, J., Xiao, Y., Tian, T., Xu, F., Hong, X., and Zhu, M.X. (2020). TRPC channels: Structure, function, regulation and recent advances in small molecular probes. *Pharmacol Therap* 209**,** 107497.

Woo, J.S., Cho, C.-H., Kim, D.H., and Lee, E.H. (2010). TRPC3 cation channel plays an important role in proliferation and differentiation of skeletal muscle myoblasts. *Exp Mol Med* 42**,** 614-627.

Zhu, M.X. (2005). Multiple roles of calmodulin and other Ca^2+^-binding proteins in the functional regulation of TRP channels. *Pflügers Archiv - Eur J Physiol* 451**,** 105-115.
